# The reach of fertility decline: a longitudinal analysis of human capital gains across generations

**DOI:** 10.1186/s41118-022-00176-4

**Published:** 2022-10-07

**Authors:** Stephanie M. Koning, Alberto Palloni, Jenna Nobles, Ian Coxhead, Lia C. H. Fernald

**Affiliations:** 1 University of Nevada, Reno, USA; 2 University of Wisconsin-Madison, Madison, USA; 3 University of California, Berkeley, USA

**Keywords:** Population and development, Fertility decline, Family planning, Birth intervals, Interpregnancy intervals, Child health and development, Intergenerational transmission, Human capital, Education, Demographic dividend, Indonesia

## Abstract

The impact of fertility decline on economic development remains central to population studies. Recent scholarship emphasizes parental investment in education as a mediator. We further develop the theoretical foundation, and empirical evidence, for the role of child health—specifically how fertility changes promote children’s physical and cognitive development and thereby complement human capital accumulation through educational gains. We test this using a two-generation model applied to Indonesian longitudinal data from 1993 to 2015. Characteristics of modern fertility regimes—older maternal ages, longer interpregnancy intervals, and lower average birth orders—generally benefit offspring cognitive development and schooling. We estimate that family planning expansion, and the resulting shift in fertility traits, induced an average increase of 0.34 years of offspring educational attainment by age 18 years. Maximal maternal educational and family planning expansion would jointly produce a 1.12-year gain, including 0.20 years more directly attributable to fertility shifts. Evidence is strengthened in parallel simulations from models of within-mother shifts, in which fertility shifts resulted in a 0.16-year gain in offspring schooling. Findings contribute new evidence for the rounding effects of women’s education and family planning expansion on human capital formation through child health within families and across generations.

## Background

The relationship between fertility declines and economic development is an enduring line of inquiry in population studies. The effects of fertility decline on economic growth were most thoroughly explored in the 1960’s and 1970’s through classic models of macroeconomic–population relationships (Coale & Hoover, 1958; Kuznets, 1960; Lewis, 1954; Solow, 1956). More recently, this line of research reemerged in scholarship describing the “demographic dividend” that could be triggered following an initial fertility decline in low- and middle-income countries (Bloom & Williamson, 1997; Bloom et al., 2000, 2003; Sanchez-Romero, 2013; Williamson, 2013). The gist of the argument is that fertility decline induces large short- and long-term shifts in age structure and these, in turn, lead to rearrangements of economic resources, labor force composition, national savings rates, and capital accumulation (Ashraf et al., 2013; Lee & Mason, 2011) as well as to advantageous micro-level shifts, such as child survival and schooling investments.

The magnitude (and duration) of the demographic dividend has been linked to three mechanisms. The first is related to changes in aggregate age composition and, in particular, to the extent to which increases in the fraction of economically active population that results from fertility decline is efficiently mined to generate human capital investments through expansion of compulsory schooling and public spending on education, e.g., physical outlays and infrastructure, and supply of qualified teachers (Duflo, 2001, 2004; Lee & Mason, 2011; Williamson, 2013). The second mechanism depends on the impetus that family size reductions can impart to systematic intra-household quality–quantity trade-offs benefiting members of less numerous birth cohorts entering the labor force two decades after the onset of the fertility decline, as they can compete in less congested labor markets (Lee & Mason, 2011; Williamson, 2013). Advantages to the first reduced birth cohort will accumulate across generations as fertility decline in one generation increases per child health and education investments (human capital concentration) in the next (Becker, 1965; Becker & Tomes, 1976).

The third mechanism is related to the very forces that promote fertility decline. Many studies show that, jointly or separately, expansion of education and increased reach of family planning programs fueled the bulk of fertility changes observed in the 1970s–1990s (Bongaarts & Watkins, 1996; Caldwell, 1980; Cleland & Wilson, 1987). These expansions tighten the association between fertility decline and increases in female (more than male) educational attainment and result in additional educational improvements in subsequent generations (Grant & Behrman, 2010). Thus, the initial fertility decline itself augments human and material resources that expand educational opportunities in subsequent generations microeconomically (i.e., increased parental investments) and macroeconomically (i.e., in infrastructure outlays and human capacity) (Acemoglu, 2010; Ashraf et al., 2013; Bloom et al., 2009; Joshi & Schultz, 2013).

Although this body of research on the demographic bonus elucidates distinct and novel pathways through which fertility limitation can enhance human capital formation and promote aggregate economic growth, it, such as the classic literature in the area, pays scant attention to a mechanism that links fertility reductions, child health, and human capital formation. This mechanism effectively adds a path through which households and families could augment “child quality effects” (e.g., Ashraf et al., 2013), or “quality–quantity tradeoffs” (e.g., Williamson, 2013). Increases in child quality occupy an important place in the demographic bonus literature but only as associated with intentional parental investments. Instead, our hypothesis emphasizes unintended health-related consequences that follow fertility declines, including better maternal health, reduced sibling competition for resources, more beneficial birth spacing, and improved quality of lactation patterns. All of these are likely to induce better child health status and physical growth trajectories. In turn, these promote increases in human capital (i.e., “quality”). We argue below that this mechanism could be as or more fundamental than those connected to aggregate age compositional shifts or intentional intrafamily changes in parental investments.

The paper is divided in two parts. In the first part we describe a stylized model that integrates two bodies of scholarship that have proceeded largely independently: one on the implications of fertility decline for early life health status and the other on the influence of early life health exposures on human capital formation. We identify pathways through which fertility limitation can enhance child health and survival and argue that family size reductions are beneficial for child health nutritional status, cognitive development, and educational attainment. These effects are distinct from but surely could magnify those invoked by the demographic dividend theory (Ashraf et al., 2013; Bloom & Williamson, 1997; Williamson, 2013).

In the second part of the paper, we assess empirical evidence to support our conjectures by deploying a multi-step analysis to describe human capital repercussions of fertility decline within families across two generations. We use Indonesian data on the health and developmental trajectories, from birth to secondary schooling completion, of birth cohorts spanning the 1990 decade.

## Part I: fertility decline, child health, and human capital formation

Several lines of research suggest that fertility reductions in one generation may have human capital returns in the next one and that these gradually accumulate over time. First, prior demographic studies show that fertility reduction has important health and survival returns through maternal health status improvements, reduction of high-risk pregnancies, reduced sibship, and more advantageous birth spacing (Bongaarts, 1987; Hobcraft et al., 1983; Palloni, 1989; Potter, 1988; Trussell, 1988). Such returns may be accrued whether initiated by family planning, educational expansions, or exogenous improvements in child health and survival. Second, a separate, newer, line of work demonstrates the importance of early child health conditions for subsequent trajectories of physical, cognitive, and emotional growth, and development of labor market skills during early childhood and adolescence (Cunha & Heckman, 2009; Heckman, 2007; Strauss & Thomas, 2007). Below, we describe relevant empirical findings from these two lines of research.

### Fertility reductions improve child health and survival

Fertility declines spurred by increases in female education and/or broader access to family planning can be the result of several altered fertility factors: stopping rules (reduction of family size), later fertility initiation (age at first birth), longer interpregnancy intervals (IPI), or any combination of these (Bongaarts, 1987; Gertler & Molyneaux, 1994; Potter, 1988; Trussell, 1988). Most of these changes have potentially significant health-related payoffs. Smaller family size and longer IPI’s consistently predict improved maternal health, increases in maternal recovery and post-pregnancy health status, and reduced-risk births across country settings and social groups (Barclay et al., 2020; Conde-Agudelo et al., 2012; Hobcraft et al., 1983; Molitoris et al., 2019; Potter, 1988; Trussell, 1988). Early fertility initiation, or young maternal age at first birth, is associated with some maternal nutritional deficiencies, including anemia, in high-income countries (Gibbs et al., 2012).

Beyond maternal and reproductive health outcomes, poorer child health outcomes are associated with younger maternal ages at birth across 55 low-to-middle-income countries, including higher risks of child mortality, stunting, wasting, diarrhea, and anemia (Finlay et al., 2011). Reduced sibship size also has documented health benefits on all household children across settings in high-income countries via decreased sibling competition and increased access to material resources and parental attention (Conley & Glauber, 2005; Downey, 1995). However, this association and how it relates to educational attainment may vary in different low- and middle-income settings, including Indonesia (Maralani, 2008).

Longer IPIs are associated with reduced maternal and child risks of illnesses, better nutritional status, and lower mortality risks (Hobcraft et al., 1983; Palloni, 1989; Rutstein, 2005). Conversely, short IPIs have been linked to maternal depletion (Winkvist et al., 1992), increased child competition for scarce material resources and parental care (Boerma & Bicego, 1992), and shortened breastfeeding (Marquis et al., 2002). These may hinder children’s subsequent physical, cognitive, and emotional growth and development (Adair et al., 2013; Brown & Pollitt, 1996; Glewwe & Miguel, 2007; Glewwe et al., 2001; Hoddinott et al., 2008; Martorell et al., 2010; Stein et al., 2010). Very recent empirical evidence from a massive study of 77 countries, at various levels of development, and 4.5 million births from Demographic Health Surveys (DHS) confirms that short birth intervals increase the risk of infant mortality (Molitoris et al., 2019).^1^ An important issue for the argument we make in this paper (see below) is whether short birth intervals also derail normal patterns of physical growth and development. It could be that short birth intervals have only a transient impact on post-natal growth trajectory that is partially offset or removed by catch-growth in later stages of development, as suggested by some recent empirical evidence (Miller & Karra, 2020). However, it is unclear whether growth offsetting is universal, beneficial, or requires the presence of environmental conditions (household and family-based traits) to be activated (e.g., Cameron & Demerath, 2002).

Finally, breastfeeding is important for early life health, physical growth, and development. It provides macro- and micro-nutrients, promotes neurological and brain development, and strengthens immune function and maternal–child bonding. Thus, the absence or very short duration of breastfeeding (less than 3 months) and inadequate supplementation increase children’s risks of poor nutrition and infection, weaken immune responses, may impair brain development, and increase mortality risks (McDade & Koning, 2020; Palloni, 1989; Schack-Nielsen & Michaelsen, 2007; Victora et al., 2008). Ultimately, these effects translate into poor physical, cognitive, and emotional development of siblings with tight birth-spacing (Goosby & Cheadle, 2009; Knudsen, 2004; Pollitt et al., 1996; Power et al., 2006; Roncagliolo et al., 1998). By reducing the incidence of very short IPIs, fertility decline expands opportunities for realizing the benefits of breastfeeding.^2^ As we argue below, these changes may all produce improvements in child nutritional status and early life physical growth and, subsequently, in cognitive and non-cognitive development (Chatterjee & Sennott, 2020; de Andraca & Uauy, 1995; Martorell et al., 2010; Pollitt et al., 1995, 1996, 1998; Rana et al., 2020; Stein et al., 2010; Uauy & De Andraca, 1995; Uauy et al., 1995; Victora et al., 2008).^3^

### Child health, physical growth, and cognitive development

A second strand of research includes strong empirical evidence demonstrating the importance of early child health on cognitive and emotional development (e.g., Glewwe & Miguel, 2007; Hoddinott et al., 2008; Martorell et al., 2010; Victora et al., 2008). Together, this large and diverse body of literature suggests direct and indirect pathways (through nutritional and health status) through which family size reductions increase children’s cognitive capabilities. Small order births have also been directly associated with greater cognition (Black et al., 2011) and strategic ‘soft skills’ (Berglund et al., 2005), although these studies were in high-income countries.

Children’s physical growth trajectories have been linked directly to their subsequent educational attainment, performance in school, and adult earnings in the labor market (Currie, 2009; Strauss & Thomas, 2008). Similarly, early nutritional status predicts cognitive development (Brown & Pollitt, 1996; Mendez & Adair, 1999; Pollitt et al., 1995), psychological well-being (Brown & Pollitt, 1996; Walker et al., 2007), social well-being, and physical development (Habicht & Martorell, 2010; Kristjansson et al., 2006). Poor nutritional status and recurrent bouts of illnesses increase school absenteeism and reduce children’ abilities to learn and develop skills and traits relevant for educational attainment and labor market performance and experience (Alderman et al., 2006; Anne Case & Christina Paxson, 2008; Glewwe et al., 2001; Hoddinott et al., 2008; Spernak et al., 2006).

Finally, other early life conditions (e.g., poverty and adversity) highly correlated with fertility have independent impacts on educational attainment and labor market success across generations in diverse settings (Cunha & Heckman, 2009; Heckman, 2007; Palloni, 2006; Victora et al., 2008). One important implication of these relations is that schooling and human capital potential are shaped very early in life and well before entering the labor market, including through positive feedback loops whereby “learning begets learning” (Heckman & Masterov, 2007). This is confirmed by research showing that economic outcomes throughout life are a function of investments made many years before and have lasting implications intergenerationally. Empirical findings suggest the existence of an intergenerational association between poverty and precarious human capital (Bird, 2007; Durlauf, 2003; Magnuson & Votruba-Drzal, 2009), whereby family backgrounds (partially determined by completed family size) constrain children’s own contribution to adult human capital formation and, indirectly, to aggregate economic growth and inequalities. These findings suggest that the ripple effects of poor early conditions and health are significant and lasting.

### Pathways linking fertility decline, child health and human capital formation

Because a secular fertility decline involves changes in stopping rules (‘fertility limitation’) and shifts toward more advantageous child spacing regimes (age of initiation, timing of births, and breastfeeding), it can influence early child physical and mental health status, child physical growth and cognitive development, and the acquisition of human capital-relevant traits in the offspring generation. Thus, we expect that investments in family planning programs that either satisfy unmet contraceptive demand or erode the foundations of traditional high fertility norms (or, likely, both) will have intergenerational payoffs, including human capital gains, upward educational mobility, and subsequent fertility reductions. Similarly, by triggering an initial fertility reduction and subsequent effects on child physical and mental health, any exogenous improvement in maternal educational attainment can generate healthier birth cohorts, greater child educational attainment, and, ultimately, greater intergenerational human capital accumulation.

In summary, the seeds of significant human capital growth are contained in initial fertility declines and include maternal and child health status amelioration. We suggest that these changes have a more direct impact on human capital formation than has been discussed in research on the macro-level effects of fertility decline, i.e., the health-related consequences. If so, increases in maternal education, adoption of family planning, or both, could initiate fertility declines that spill over to the offspring generation via acquisition of health traits and skills and higher educational attainment. Our conjecture about the influence of fertility declines on intergenerational human capital formation, intended and unintended, involves three pathways, graphically represented in Fig. 1.

## Part II: empirical evidence

### Indonesia’s fertility transition

We consider evidence for these processes in Indonesia, during a period of marked socioeconomic development. Apart from the economic crisis in 1998–1999, the Indonesian gross domestic product grew over 5% a year since 1970. This was accompanied by large expansions in the education system (Duflo, 2004; Jones et al., 2013; Yeom et al., 2002). In 1970, less than 20% of adolescents had completed primary school. By 2015, over 95% of eligible-aged children were enrolled in primary school and over 65% of eligible-aged children were enrolled in secondary school. Gains in women’s education paralleled men’s, with a now negligible gender gap in secondary school completion rates (BPS, 2012; Surbakti & Devasahayam, 2015; UNESCO, 2018).

Perhaps unsurprisingly, the 1960–2015 period included dramatic declines in fertility and family size. In 1960, the TFR exceeded 6 births per woman. By 2012, the TFR had fallen below 2.5 (Hull, 2015; Hull & Hartanto, 2009). Reductions were attributed to postponement of age at marriage and first birth among younger women, particularly those in urban areas, with peak fertility shifting from ages 20–24 years to 25–29 years by 1991 (Central Bureau of Statistics (CBS), 1998). Fertility among women under 20 years still constituted 11% of the total fertility rate in 1995–1997. Meanwhile, interpregnancy intervals (IPIs) were also documented as getting longer, with the percentage of IPIs under 24 months declining substantially. In 1991, 19.6% of births were preceded by an IPI < 24 months (National Research Council, 2013). It was 12.7% of births by 2002 and 10.7% births by 2012. During the approximate decade more closely aligned with the study birth cohort, the TFR dropped from 3.4 to 2.6 between 1987 and 2002 (National Research Council, 2013).

Declines were driven in part by gains in women’s education and labor force entry (Gertler & Molyneaux, 1994) but also supported by an aggressive stance toward family planning provisions nationwide. The National Population and Family Planning Board (*Badan Kependudukan dan Keluarga Berecana Nasional, BKKBN*) was founded in 1970 and quickly grew in size, scope, and stature (Hull, 2005). By 1979, over 2 million women acquired contraception through the BKKBN (ibid). By 1991, unmet need for contraception among fecund-age women had dropped to 17%, and by 2012 reached 11% (BPS & BKKBN, 2013).

In addition to its economic development and expansion of social services, Indonesia’s remarkable regional and ethnic diversity make it a particularly interesting case study. Geographically, the 250-million-person population is spread across 14,000 islands in an archipelago spanning 5100 km east to west. The population’s composition has been shaped by centuries of migration and occupation from multiple colonial presences (Hirschman & Bonaparte, 2012). The fertility transition has proceeded with remarkable regional heterogeneity; in 1990, province-level TFR values ranged from 2.9 to 5.0 (Additional file 1: Fig. S1). Thus, the current study cohort, born in Indonesia between 1990 and 2000, can leverage remarkably different fertility patterns across place and time to assess evidence of their enduring impacts on child health and schooling trajectories.

### Approach

The current study entails a multi-step analysis to uncover the intergenerational human capital repercussions of fertility decline within families, focusing on the health and development trajectories of a cohort from birth to secondary school completion. First, we perform staged multivariate regressions with longitudinal data to establish whether and how child health accounts for the association between fertility and child educational attainment. Second, we assess the potential impact of fertility decline by leveraging simulations that use an expanded model of the relationships between fertility, child health and development, and educational attainment. We describe the potential human capital impacts of expansive shifts in family fertility in the entire study cohort, as would be expected with further expansions to women’s education, family planning, or both. As one of our sensitivity checks, we run alternative models and simulations with maternal fixed effects that adjust for potential confounding maternal characteristics by limiting comparisons to within mothers and a subset of the fertility factors of interest. As a secondary analysis, we examine the role of breastfeeding.

### Data

We use data from the Indonesian and Family Life Survey (IFLS), a longitudinal household survey representative of 83% of the Indonesian population, to construct a cohort of household members born between 1990 and 2000 and followed from early childhood into late adolescence. Because of the spatial and social diversity in Indonesian development and the attendant pacing of fertility decline, cohort members’ household sizes range from 1- to 16-child households. Maternal fertility behaviors vary along with education, family resources, and attendant investments in children’s health. The first wave was fielded in 1993, during which members of 7224 households in 321 communities from 13 provinces were interviewed (Frankenberg et al., 1995). Follow-up waves were collected in 1997 (Frankenberg & Thomas, 2000), 2000 (Strauss et al., 2004), 2007 (Strauss et al., 2009), and 2014 (Strauss et al., 2016). Considerable effort put into tracking respondents has resulted in remarkably low attrition for a survey of this size (Thomas et al., 2012), with re-contact rates of 94%, 95%, 94%, and 92% for consecutive follow-up waves (Strauss et al., 2016).

The IFLS collects data at the individual, household, and community level, including detailed sociodemographic, economic, and health information from all household members. Fecund-age women provide fertility histories that are updated in each wave of the survey. Detailed data on conditions during pregnancy and delivery are collected on births occurring in 5 years prior to IFLS 1; this information is also updated in each survey wave.

We linked maternal and fertility characteristics to each cohort member along with longitudinal measures of child and adolescent health, cognition, and educational attainment. Births eligible for inclusion were those reported by an IFLS mother within 3 years after birth in wave 1, 2, or 3. Of the 6627 eligible births, 62% were retained with health, cognitive, and education measurements recorded in at least three subsequent waves. Of those, 93% had non-missing data and were included in the final analysis. Complete cases (Table 1) and all eligible births (Additional file 1: Table S1) did not substantially differ in fertility, maternal education, child health, or other measured characteristics. Due to the study’s complex estimation strategy, missingness being concentrated among outcome variables, and minimal differences between complete and incomplete cases, a complete case analysis was conducted to avoid bias related to imputation model misspecification and inefficiency (Hughes et al., 2019; Sterne et al., 2009).

### Measures

Descriptive statistics are shown in Table 1.

Our fertility indicators were specific to each index child, using cutoff values that would allow us to model and simulate shifts toward more family planning, away from high fertility (higher birth order, younger maternal ages, and short IPIs), that have been associated with improved maternal and child health. First, exposure to family planning education, and access, was measured as a single binary indicator of whether women reported knowledge of all four of the following contraceptive methods: contraceptive pills, intrauterine devices, contraceptive injections, contraceptive tubes. Second, we used three categories for birth order. We categorized first births separately to stratify by first and non-first births, given the difference in relevant fertility factors (e.g., first births do not have a pre-pregnancy interval) and varied impacts of fertility (e.g., the association between maternal age and birth weight differs by birth order). We categorized second births separately from higher order births to prepare for simulating declines to replacement fertility, which Indonesia has been trending toward (Hull, 2015; Hull & Hartanto, 2009), including during the birth cohort period studied (Additional file 1: Fig. S1).

Third, for maternal age, we used ages 20 years and younger as indicative of both a relatively high-fertility regime and a risk factor for poor child health and growth in low- and middle-income countries (Finlay et al., 2011). Fourth, short IPIs were measured based on self-reported maternal pregnancy histories, and included separate measures for pre-pregnancy and post-birth IPI durations less than 24 months, based on the latest World Health Organization (WHO) guidelines (World Health Organization, 2006). Finally, the total number of children 18 years and younger in the household was included as an additional continuous covariate at the time of each child outcome measure that, beyond birth order, is indirectly associated with fertility, child health, and education outcomes. While not considered part of the direct interventions simulated in the family planning and education expansion scenarios, this variable was set to be consistent with each simulation (e.g., set to 2 for low fertility scenarios). Maternal education was categorized by years completed (< 6, 6, 7–11, or ≤ 12).

Our earliest child health measure was birthweight (continuous in kilograms). Height-for-age measurements were *z*-scores based on sex- and age-specific median height values in a well-nourished population created by the WHO (Onis et al., 2007). Cognition for each child was measured using age-standardized scores from a two-part assessment: (1) a shape-matching exercise from the Raven’s test of fluid intelligence, and (2) a numeracy test; both tested and validated in Indonesia (Strauss et al., 2016).

We measured children’s human capital accumulation using school years completed by 2014 (IFLS 5) when the children were 14–24 years. Compulsory education in Indonesia comprises 12 years of schooling, which is typically completed by the age of 17–18 years. Since not all individuals had completed schooling at the time of measurement, years were standardized by age to achieve comparable measures of educational attainment by adolescence or early adulthood. Models adjusted for covariates measuring birth year (continuous), maternal cognition (*z*-scores constructed from same measures as children), and binary indicators for urbanicity, child sex, multiple gestation births, and maternal marital status at birth (1 = married; 0 = not married).

### Model

Our approach aims to incorporate as many key channels through which health-related advantages of a modern fertility regime translate into elevated human capital formation as possible while also attending to maternal education as an upstream factor influencing fertility (Fig. 1). To start, we use province fixed-effects regressions to establish that family fertility indeed predicts offspring educational attainment, accounting for time-invariant environmental factors at the provincial level and adjusting for additional maternal and child characteristics. We additionally stratify by birth order, running models separately for first births and non-first births, to account for underlying differences in associations by parity and variable fertility parameters (e.g., preceding IPI is not estimable among first births). Second, we confirm that accounting for child health indeed attenuates this association, suggestive of mediation.

Next, we expand the above models to define associations of interest in a system of non-recursive equations, or structural models, that form the foundational model set for our scenario simulations, as detailed further below and in prior studies deploying this approach (Palloni, 2006; Palloni et al., 2009). We use mother–child pair data spanning each child’s birth (t=0), childhood over multiple timepoints from ages 0–13 years (t=1,2,3), and educational attainment by age 14–24 years (t=4). Each equation below relates outcomes measures longitudinally over time $\left(t\right)$ to fertility and health predictors at the index child level $\left(i\right)$, with fixed effects to account for unobserved time-invariant hetero-geneities across province $\left(j\right)$:

(1) $$B_{ij(t=0)}=\alpha_{0j}+\alpha_{v}V_{ij(t=0)}+e_{Bij}$$

(2) $$S_{ij(t=1)}=\beta_{0j}+\beta_{w}W_{ij(t=1)}+es_{1ij}$$

(3) $$S_{ij(t=2)}=\gamma_{0j}+\gamma_{x}X_{ij(t=2)}+es_{2ij}$$

(4) $$C_{ij(t=3)}=\delta_{0j}+\delta_{y}Y_{ij(t=3)}+e_{Cij}$$

(5) $$E_{ij(t=4)}=\zeta_{0j}+\zeta_{z}Z_{ij(t=4)}+e_{Eij}$$

where $B_{ij}$ is birthweight; $S_{ij}$ is physical growth; $C_{ij}$ is cognitive ability; and $E_{ij}$ is educational attainment. Characteristics defined before birth are included in the vector, $V_{ij(t=0)}$, including maternal fertility measures (except for post-birth IPI), maternal education, and covariates (presented above in Measures and listed in Table 2 and Additional file 1: Table S2). The vector of coefficients predicting birth weight is indicated by $\alpha_{v}$ and the province-specific intercepts are denoted with $\alpha_{0\text{j}}$. Idiosyncratic errors are included in the e terms, which encompass variation unexplained at both the individual- and province-level. The remaining equations are to be interpreted similarly, with outcome-specific intercepts and coefficients denoted in Eqs. (2) through (5) by β, γ, δ, and ζ. Each equation includes predictors from preceding equations, along with additional predictors corresponding to the progressive timing of each outcome, as indicated by $W_{ij(t=1)}$, $X_{ij(t=2)}$, $Y_{ij(t=3)}$, and $Z_{ij(t=4)}$. For example, $W_{ij(t=1)}$ adds the post-birth IPI duration and updates the total number of children in the household to t=1. In addition, equation coefficients incorporate the lagged values for the outcomes in preceding equations, e.g., $B_{ij(t=0)}$ is included in $W_{ij(t=1)}$. Additional file 1: Table S2 further details estimation equations and terms.

### Cumulative impacts

To evaluate the cumulative impact of fertility changes on human capital in the offspring generation, we use the above estimates to simulate a series of counterfactual scenarios. These are meant to reflect fertility changes initiated by possible expansions in either maternal education, family planning services, or both.

We start with a set of background traits for each individual i in province j, contained in vector $V_{ij(t=0)}$. This vector of initial characteristics and the estimated parameters of the first equation yields predicted values in $B_{\mathrm{ij}(t=0)}$, to which we add an idiosyncratic normally distributed error with mean zero and standard deviation (SD) equal to the square root of the estimated mean square error. Values are then included in $W_{ij(t=1)}$ to predict $S_{ij(t=1)}$, with simulated random error. We proceed accordingly until we assign outcome values,$E_{ij(t=4)}$. We replicate simulations 500 times, which generates outcome distributions instead of single mean values.

To assess the impact of fertility declines on children’s educational attainment through child health and cognition, we simulate counterfactual changes in maternal education and fertility, both historical focuses of development-related interventions. We then compare the outcome distributions across scenarios, in relation to the baseline without any manipulation (*A*). While maternal education and fertility are not entirely separable, we design the simulations below as an initial step to describe their relative impact and to inform future work with a capacity to take an even more mechanistic approach.

- *Exogenous change in family planning services (B)* For this scenario, we simulate family fertility changes that reflect an exogenous increase in the availability and use of family planning services, unrelated to (or in the absence of) shifts in maternal education. We assign low fertility indicators to all births: (1) high maternal contraceptive use knowledge; (2) maternal age of > 20 years; (3) longer IPIs; and (4) fertility (parity = 2). We interpret the difference between this simulation’s results and our baseline as indicative of the potential impact of sweeping family planning expansions during the study period, through the child health improvements measures, on individual’s human capital accumulation. Here, we simulate results separately for first and non-first births, using the stratified analyses and respective samples. Then, we assess stratified results and aggregate results with first and non-first births weighted equally to estimate mean outcome distributions at parity = 2.
- *Exogenous changes in maternal education (C)* For this scenario, we simulate fertility changes that we would expect to occur *through* increased maternal education, specifically a universal increase to 12 or more years of educational attainment. This aligns with an increase in public education spending that augments infrastructure and improves the supply and quality of teachers (assuming there is unmet demand for education and that the family costs of sending children to school remain invariant). Then, at least one cohort of women would experience a shift toward more years of schooling and, as a result, we would expect the fertility-related factors among these mothers would shift. To assess how, along with manipulating maternal education, we also manipulate the distribution of fertility characteristics to match those of women with ≥ 12 years of schooling in observed our sample. These distributions are summarized in Additional file 1: Table S2. Then, we assess results as stratified by birth order and as aggregate means weighted by the observed distribution of first and non-first births among mothers with ≥ 12 years of schooling.
- *Exogenous changes in family planning services and maternal education (D)* This scenario combines (*B*) and (*C*), with maximal adjustments to maternal education and fertility jointly. Its comparison with (*B*) and (*C*) allow us to assess the relative contributions of fertility and maternal education, respectively, to cumulative impacts. We assess birth order-stratified results and aggregate mean distributions weighted to equate parity = 2.

### Secondary analyses and sensitivity checks

To address endogeneity concerns, specifically unmeasured maternal characteristics that contribute to both fertility and child outcomes, we ran multiple sensitivity checks with added controls. This included rerunning original models with an additional indicator variable included for any child or pregnancy losses prior to outcome measurement (single time-variant binary indicator in each health outcome model). A second set of sensitivity checks involved a single model with maternal fixed effects to isolate within-mother comparisons, including resulting scenarios under baseline and low fertility (education comparisons irrelevant due to no within-mother variation).

To investigate breastfeeding as a potential explanation of the fertility–health relationship, we also ran an alternative set of regressions with breastfeeding duration added to evaluate any changes in coefficients across outcomes that may indicate mediation.

### Results I: model estimates

Our initial adjusted regression supported our expectation that higher fertility circumstances predict lower educational attainment in adolescence across birth orders (Additional file 1: Table S4). The most consistently strong association was captured specifically in the adjusted association between more children in the household and lower attainment. Among first births, young maternal age was additionally a strong predictor of lower attainment. Among non-first births, short IPI’s were additionally associated with lower attainment. Other fertility-related coefficients were in consistent directions but not statistically significant. Adjusting for child health indicators measured between birth and adolescence resulted in an attenuation of associations above, and the association between maternal and offspring education. For example, among first births, adjusting for child health attenuated the education outcome coefficients for young maternal age and number of children in the household by 8.4% and 10.1%, respectively. In contrast, coefficients for contraceptive knowledge and pre-pregnancy interval did not attenuate. Among non-first births, coefficients were more substantially attenuated for young maternal age (33.8%), pre-pregnancy interval (36.3%), birth order ≥ 3 (11.7%), and number of children in household (7.2%). In our more conservative maternal fixed effects models (i.e., sibling comparisons), coefficient attenuation after accounting for child health occurred specifically for maternal age (11.3%), pre-pregnancy interval (47.7%), and number of children in household (69.9%).

With this motivating set of associations in hand, we turned to the expanded regressions that included each intermediate outcome, revealing underlying relationships further. Most coefficient estimates relating fertility characteristics to child health and cognition outcomes operated as expected, with higher fertility-related factors negatively associated with child health outcomes. Among first births (Table 2), maternal ages ≤ 20 years were significantly associated with lower early growth. Among non-first births (Table 3), young maternal age and short pre-pregnancy intervals consistently predicted worse child health outcomes. Unexpectedly, high contraceptive knowledge was negatively associated with growth at *t*_1_. Birth orders of three and higher were significantly and positively associated with birth weight. Also, having more household children was associated with growth at *t*_2_ conditional on earlier growth, i.e., catch-up growth. Maternal fixed effects model coefficients followed consistent patterns.

Tables 2 and 3 also include child health coefficients predicting lagged child health outcomes and educational attainment. Among first births, child health predictors are strongest for proximate health outcomes, with only cognition significantly predicting educational attainment. A one-kilogram increase in birth weight predicted a 0.510-SD increase in height-for-age at *t* = 1 (*p* < 0.001), a 1-SD increase in height-for-age at *t* = 1 predicted a 0.846-SD increase in height-for-age at *t* = 2 (*p* < 0.001), a 1-SD increase in height-for-age at *t* = 2 weakly predicted a 0.0463-SD increase in cognition-for-age at *t* = 3 (*p* > 0.05), and a 1-SD increase in cognition-for-age at *t* = 3 predicted a 0.159-SD increase in education-for-age at *t* = 4 (*p* < 0.001). Among non-first births, these same associations were similarly strong, with birth weight also significantly predicting height-for-age at *t* = 2, adjusted for height-for-age at *t* = 1. Maternal fixed effects model estimates were also comparable.

### Results II: cumulative impact simulations

The simulated exogenous shifts to fertility characteristics and maternal education each stimulated significant cascading shifts in child health and offspring education, as shown in aggregated results from parity-stratified models and simulations. Figure 2 summarizes outcome distributions for each scenario, across 500 replicated simulations. Shifts to expansive family planning included later fertility initiation, from 12.6% to 0% maternal ages ≤ 20 years at birth; longer IPI’s, from 9.7% to 0% pre-pregnancy intervals < 24 months and from 13.8 to 0% post-birth IPI’s < 24 months; and fewer births, from 41.5% to 0% birth orders ≥ 3. Shifts toward maximal maternal educational expansion meant manipulating upper secondary school (all 12 years of compulsory schooling) completion from 27.9% to 100% among cohort mothers.

Relative to baseline estimates, simulations representing the expansion of family planning and the resulting shift in traits of the fertility regime alone (*B*) induced an average increase of 0.34 years by age 18 years. In comparison, universal maternal education expansion to at least 12 years of schooling and attendant shifts to fertility (*C*) led to an average increase in offspring’s education of 0.92 years. Joint maximal expansion of maternal education and family planning (*D*) increased offspring education by 1.12 years. The difference in means between (*C*) and (*D*) represents the estimated net influence of fertility when maximal maternal education and family planning coincide: 0.20 years. Additional summary outcome distributional shifts under each scenario are included in the supplement (Additional file 1: Table S5), as are parity-stratified simulation results (Additional file 1: Fig. S2). Adjusting for prior maternal pregnancy or child loss did not substantially alter coefficients (Additional file 1: Table S6).

### Sensitivity check: maternal fixed effects

Maternal fixed effects models resulted in mostly consistent estimates, as expected, including the attenuation of fertility coefficients predicting education when child health measures were added (Table 4 and Additional file 1: Table S7). In within-mother comparisons, the negative association between young maternal age and educational attainment was exceptionally strong, independent of birth order. Some of the unexpectedly strong associations in the original non-first birth models did not appear when fixed effects among all births were deployed. For instance, birth orders of 3 and higher no longer positively predicted birth weight. Simulations from the fixed effects model (Fig. 3 and Additional file 1: Table S8) estimate that downward fertility shifts would result in an upward shift in mean educational attainment by 0.07 SD, or 0.16 years at age 18. When predicted separately for first and second births, cumulative educational improvements are specifically among first births.

### Secondary analysis: the role of breastfeeding

Additional file 1: Table S9 shows secondary analyses more closely examining the role of breastfeeding. Among first births, breastfeeding duration is negatively associated with more children in the household and short post-birth pregnancy interval, as expected. Among non-first births, breastfeeding duration is negatively associated again with short post-birth pregnancy interval and maternal experience of a prior fetal or child death. It is also negatively associated with birth year and urbanicity across birth order. As expected, longer breastfeeding duration is associated with higher cognition. Surprisingly, it also predicts lower height at *t*_1_ and does not attenuate most associations between fertility characteristics and child health or education as originally expected.

## Summary and discussion

Demographic literature has historically tied fertility decline to economic growth through shifting age structures, more concentrated household investments in, and greater capital opportunities for, less dense cohorts, and positive feedback loops between lowered fertility and educational expansion (Duflo, 2001; Grant & Behrman, 2010; Lee & Mason, 2011; Williamson, 2013). Our paper extends classic approaches to further incorporate household- and individual-level mechanisms linking fertility reductions, child health, and human capital accumulation. It thereby contributes greater understanding to whether and how overlooked micro-level mechanisms, particularly child health, account for the micro- and macro-level mechanisms more commonly documented across development contexts, e.g., “child quality effects” (e.g., Ashraf et al., 2013). Our novel approach drew from a population-based longitudinal cohort from Indonesia, born over a decade of sharp fertility decline in an informative low-to-middle-income context. Evidence suggests that child health improvements likely augment other household-level investments to produce human capital gains, i.e., educational attainment.

Our baseline models are consistent with past studies documenting the association between fertility reduction and child health improvements, and between child health improvements and educational attainment. First, we uncover sizeable associations between cumulative fertility patterns and child health (detailed below), and individual associations between select fertility factors and health outcomes. Among first births, young maternal age at birth predicted stunted early physical growth, similar to its prediction of stunting and wasting in comparable development contexts (Finlay et al., 2011). Among non-first births, shorter pre-pregnancy interval predicted stunted early growth, also comparable to other development contexts (Fink et al., 2014) and plausibly related to maternal health mechanisms identified across diverse settings (Barclay et al., 2020; Conde-Agudelo et al., 2012; Hobcraft et al., 1983; Molitoris et al., 2019; Potter, 1988; Trussell, 1988). Young maternal age at birth also predicted lower cognition scores in adolescence, a finding for which we could not find comparable past studies in development contexts but likely indicates complex social and behavioral mechanisms warranting further investigation (Chittleborough et al., 2011; Shaw et al., 2006). While maternal fixed effects model coefficients were smaller, most fertility coefficients operated in expected directions, particularly the strong negative association between pre-pregnancy interval and early stunted growth. Second, our models capture human capital associated with cumulative health benefits, agreeing with a wide and diverse body of literature focused on early health trajectories and later educational and economic gains (e.g., Cunha & Heckman, 2009; Heckman & Masterov, 2007; Strauss & Thomas, 2007). Proximal health indicators formed significant predictive chains from birth weight to child and adolescent growth to cognition and, finally, educational attainment.

We additionally found, across birth orders, evidence of child health improvements linking fertility reduction to human capital. This was supported by the partial attenuation of multiple fertility coefficients predicting years of schooling after adjusting for child health in parity-stratified and maternal fixed effects models. Specifically, adjusting for child health substantially attenuated education outcome coefficients for young maternal age and number of household children across birth orders, along with pre-pregnancy IPI and high birth order among non-first births. Findings were consistent in our more conservative, non-stratified maternal fixed effects models (i.e., sibling comparisons), in which maternal age, pre-pregnancy IPI, and total household children coefficients attenuated after accounting for child health. These findings comprise a key contribution of the current study in bridging prior fields focused either on fertility and health alone, or health and education alone.

Our adaptable structural model approach, paired with simulation techniques, further revealed the contribution of aggregated and expansive fertility reduction on child health (Figs. 2, 3, Additional file 1: Table S5, and Table S8). For example, simulated shifts toward expansive family planning, i.e., lower fertility, were associated with aggregate (first and non-first births combined) shifts of 0.09 SD in height (*t*_1_), 0.10 SD in height (*t*_2_), and 0.02 SD in cognition (*t*_3_) (Additional file 1: Table S5). The scenarios shifting fertility factors between siblings alone (from the maternal fixed effect model) toward expansive family planning resulted in upward shifts of 0.22 SD in height (*t*_1_), 0.29 SD in height (*t*_2_), and 0.09 SD in cognition (*t*_*3*_) (Additional file 1: Table S8). In sum, this evidence suggests a modest, yet nontrivial, role of health and development contributing to human capital, which agrees with other macrolevel studies in development contexts (Ashraf et al., 2008).

While our approach did not produce magnitudes of association directly comparable with past demographic dividend studies, it produced comparisons between intervention scenarios relevant to Indonesia during the study period, and development contexts broadly. We compared our cohort baseline of observed values with alternative potential outcomes when fertility and education were manipulated, along with naturally cascading child health improvements. In doing so, we offer an internal comparison between manipulating fertility patterns aligning with family planning and manipulating female educational expansion, a well-established predictor of intergenerational health and education. We estimated impacts of maximal family planning expansion, including shifts toward more health advantageous maternal ages (Finlay et al., 2011), compliance with current WHO guidance on IPI’s (World Health Organization, 2006), and parity/birth orders aligned with replacement fertility, which Indonesia has been approaching since the beginning of its fertility transition (The World Bank, 2022), and the study cohort birth period (Additional file 1: Fig. S1). In our simulations, these shifts cumulatively induced an average increase of 0.34 years of offspring educational attainment by age 18 years.

Educational gains resulting from fertility reduction alone represent 10% of the 3.4-year increase in young adult education (ages 20–30 years) among Indonesians between 1993 and 2014 (Beatty et al., 2021). When combined with maximal maternal educational expansion, attainment would rise by 1.12 years, 0.20 years (17.9%) of which would still be attributable directly to fertility shifts. Evidence is further strengthened in parallel simulations of within-mother (between-sibling) shifts aligned with family planning expansion alone that result in a 0.16-year gain in child schooling. However, this latter estimate remains constrained by a more limited sample of mothers with multiple births in the sample and analyses that do not account for varied implications of first and non-first births—thus offering a more conservative estimate of the broad impacts of family planning expansion.

Beyond cumulatively substantial impacts of all fertility study measures combined on educational attainment, results revealed some outstanding individual associations between high-fertility factors and lower offspring educational attainment, adjusting for confounding household and province characteristics. Number of children in the household during adolescence and younger maternal age at birth (among first births specifically) both significantly predicted educational attainment, with and without adjustments for child health. This suggests that beyond child health mediated impacts measured, these factors also represented additional unmeasured child health or household mechanisms, possibly “child quality effects” (Ashraf et al., 2013), or both.

We did not find evidence of breastfeeding playing an influential role in the association between fertility and offspring education (Additional file 1: Table S9). Residual confounding may be responsible if unobserved household characteristics contributed to child health advantages correlated with formula use, positive impacts of maternal education and lower fertility were offset by breastfeeding barriers (e.g., maternal labor force participation), or both—as observed in Indonesia’s development (Lo Bue & Priebe, 2018). Still, breastfeeding was positively associated with cognition, as expected.

Importantly, our analyses estimate modest education and fertility effect sizes, since the baseline draws from observed values in the study sample born in Indonesia in the 1990–2000 decade and shifts these distributions to represent later birth cohorts, starting in the 2000’s. Given that the study cohort was already realizing female education and family planning expansions, study estimates thus represent a lower bound of the total effects of educational and family planning expansions on human capital accumulation in Indonesian overall. Data missingness—loss to follow up, unreported pregnancies, and item nonresponse—may have also weakened our estimates. While differences between complete and incomplete cases were small, individuals with missing values had slightly worse child health measures. Other missing values due to undetected miscarriages or survival bias would have further deflated estimates. In addition, due to data constraints, our model specifications involve only a subset of all relevant dimensions of the fertility regime. We do not account for other characteristics that may also respond to fertility conditions and have implications for child health and education, such as maternal health status.

This paper has several shortcomings. First, we presented a stylized and static model that captures effects of changes in only a handful of pertinent traits (contraceptive knowledge, fertility timing, quantity, and spacing). Second, the counterfactual scenarios are also simplified and only coarsely approximate outcomes in the real world, all of which involve complicated changes of the joint distributions of many characteristics. Thus, when we assume an exogenous shift in family planning uptake, we ignore effects of macro level changes that facilitate or impede educational progress and that may occur concurrently with changes in family planning. Third, we did not explicitly model the potentially large influence of regional heterogeneity. Shifts in family planning may be region-specific and the expansion or contraction of public-school funding may depend on the idiosyncrasies of local politics and administration. Regional heterogeneity in initial conditions is important.

Because of our desire to avoid inferential traps, we implemented multiple robustness checks that require simplified models that hinder nuanced population-based inferences. Our sensitivity checks, including maternal fixed effects, support our findings yet still warrant future investigation using additional causal designs to investigate how fertility patterns shape other maternal and child health measures and intergenerational capital at the household level. This includes studying the potentially varied role of breastfeeding in development contexts. For future consideration, a stronger model with greater statistical power would investigate interactions between changes in public school and parental investments in children’s education, account for potential mortality selection, include time varying assessment of maternal health status at the time of birth (and maternal depletion), refine measures of infants’ initial health status beyond birthweight, and assess breastfeeding as a moderator.

Overall, our analysis contributes a unified and adaptable approach to estimating population-level effects of individual and family-level processes—fertility decline, early life physical growth and development, and intergenerational human capital formation—typically explored in more disconnected lines of inquiry or aggregated data analyses. In so doing, we strengthen the foundation of empirical work on how improvements to women’s education and family planning have rounding effects on human capital formation in the next generation. Explicitly linking scholarship on aggregate demographic change with the burgeoning literature on the life-course effects of early life health has promising implications for the study of economic development in low- and middle-income countries.

## Supplementary Material

Supplementary Material Additional file 1: Supplementary Tables and Figures.

Supplementary Information

The online version contains supplementary material available at https://doi.org/10.1186/s41118-022-00176-4.

## Figures and Tables

**Fig. 1:**
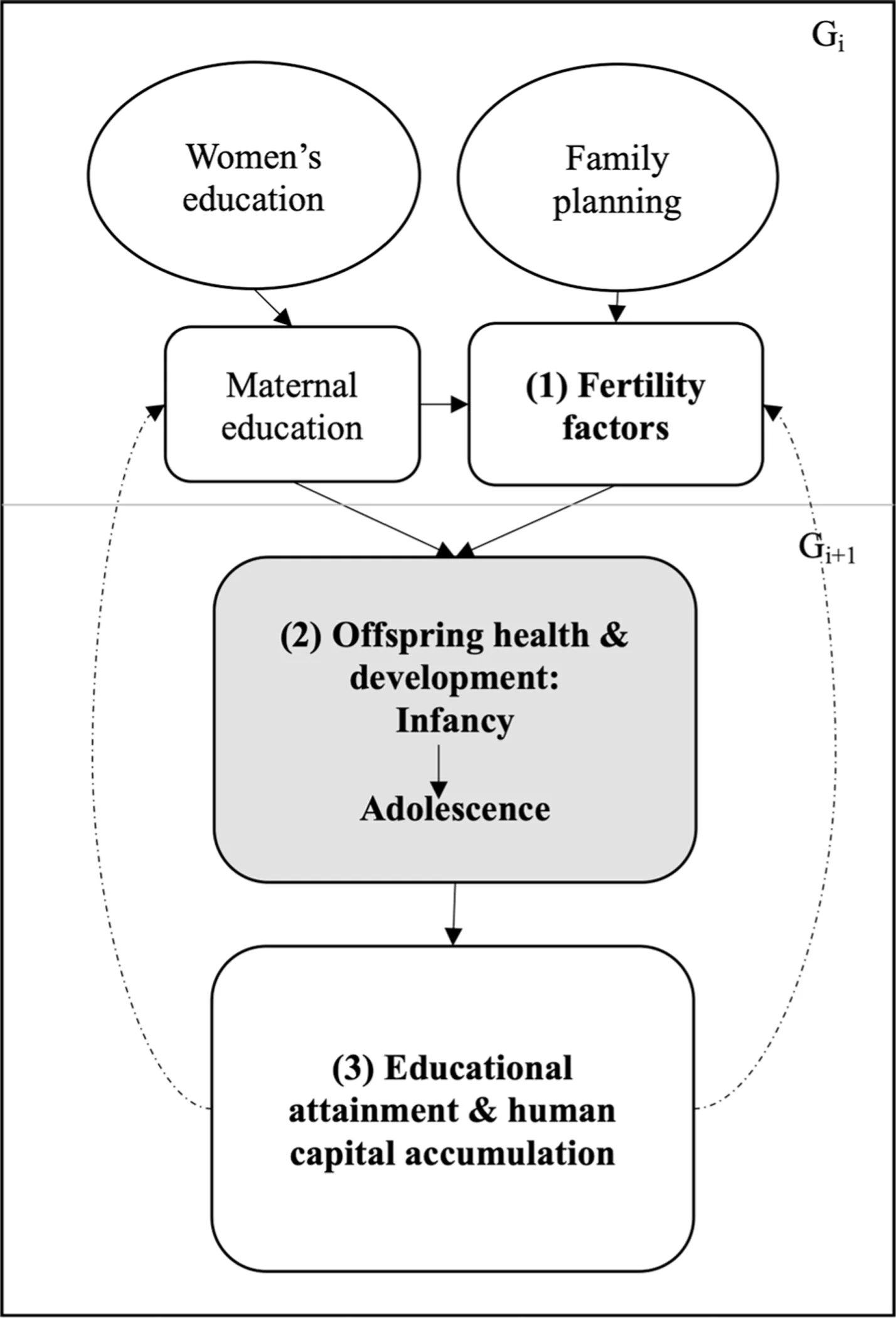
Conceptual model. Intergenerational linkages between women’s education and family planning interventions, fertility decline, child health and development, and human capital accumulation

**Fig. 2:**
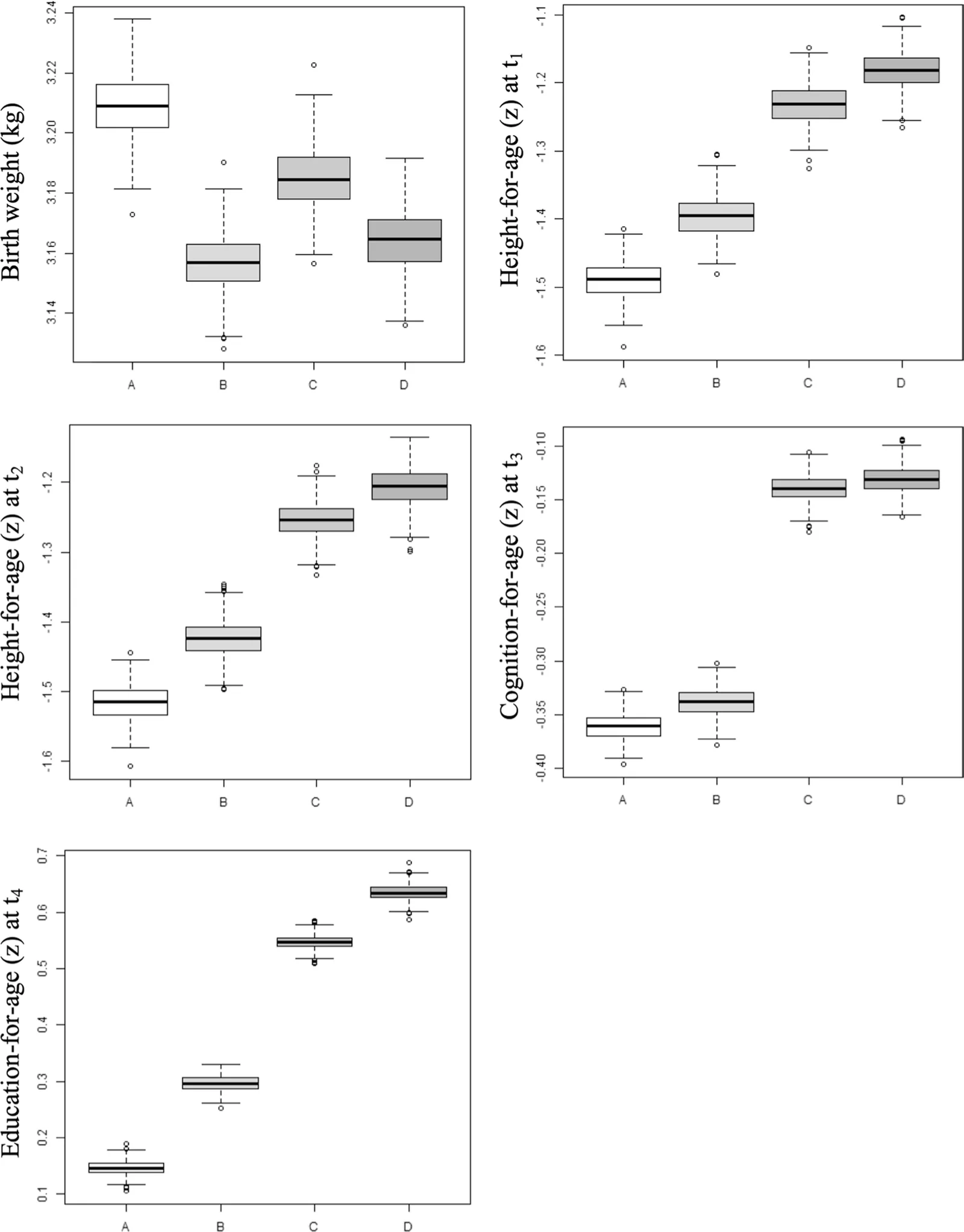
Simulated counterfactual scenarios: (**A**) baseline (no manipulation); (**B**) exogenous change in family planning services (low fertility regime); (**C**) exogenous shift in maternal education (including intermediate fertility shifts); (**D**) scenarios (**B**) and (**C**) combined

**Fig. 3:**
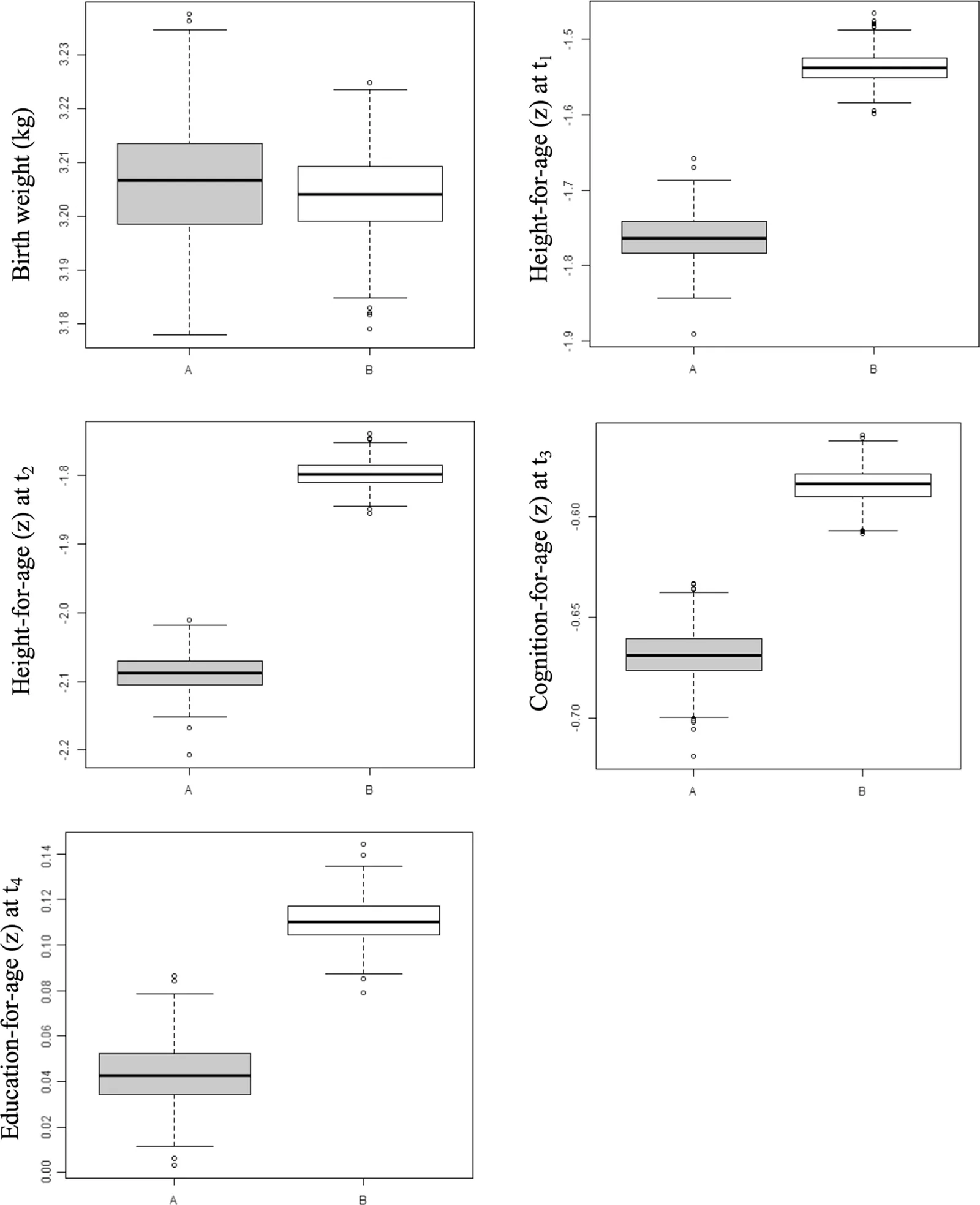
Simulated counterfactual scenarios from maternal fixed effects models: (**A**) baseline (no manipulation) and (**B**) exogenous change in family planning services (low fertility regime)

**Table 1:** Maternal and individual characteristics for cohort born between 1900 and 2000

	Mean or percent

Maternal characteristics	
Maternal highest education level	
Less than primary	21.6%
Primary	26.7%
Some secondary	23.2%
Upper secondary and above	28.5%
Maternal cognition (*z*)	0.176
Contraceptive knowledge high	79.2%
Total live births	
1	18.0%
2	40.5%
3	20.2%
≥ 4	21.3%
Mother married at birth	89.4%
Observations	1503
Birth characteristics	
Urban	44.9%
Male	51.9%
Fertility characteristics	
Mother ≤ 20 years at birth	11.5%
Multiple gestation birth	1.5%
Birth order	
1	33.6%
2	25.7%
3+	40.6%
Number of children (< 18 years) in household at birth	2.638
Pre-pregnancy interval < 24 months	9.4%
Post-birth pregnancy interval < 24 months	13.2%
Child health characteristics	
Birth weight (kg)	3.208
Height (*z*) *t*_1_	−1.489
Height (*z*) *t*_2_	−1.514
Cognition (*z*) *t*_3_	−0.360
Years of educational attainment (2014)	9.949
Observations	3678

Source: Indonesian Family Life Survey Waves 1–5

**Table 2:** Linear regression model coefficient estimates (standard errors) among first births

	Birthweight (kg)	Height *t*_1_	Height *t*_2_	Cognition *t*_3_	Education *t*_4_

Maternal educational attainment					
Less than primary	−0.0879	−0.0699	−0.0835	−0.414***	−0.417***
	(1.43)	(0.40)	(1.10)	(5.40)	(5.83)
Primary	−0.0690	−0.0209	−0.0993	−0.181**	−0.233***
	(1.50)	(0.16)	(1.76)	(3.17)	(4.40)
Some secondary	Ref.	Ref.	Ref.	Ref.	Ref.
Upper secondary and above	−0.0328	0.272*	0.0671	0.102	0.176***
	(0.77)	(2.26)	(1.28)	(1.92)	(3.59)
Maternal cognition (*z*)	0.00679	−0.0108	0.00703	0.0630*	0.0634*
	(0.28)	(0.16)	(0.24)	(2.09)	(2.28)
Male	0.0216	−0.0575	0.0130	−0.0458	−0.117**
	(0.67)	(0.63)	(0.33)	(1.15)	(3.17)
Twins +	−0.450**	−0.825*	−0.0290	0.148	0.130
	(3.18)	(2.04)	(0.16)	(0.84)	(0.79)
Birth year	−0.00385	0.0659***	0.0155*	−0.000913	−0.0245***
	(0.65)	(4.34)	(2.35)	(0.14)	(3.57)
Urban	−0.0281	0.0981	0.0489	0.104*	−0.00839
	(0.79)	(0.97)	(1.11)	(2.35)	(0.20)
Maternal marital status	−0.0237	−0.139	−0.0936	0.155	0.131
	(0.55)	(0.43)	(0.94)	(1.70)	(1.84)
Fertility characteristics					
Contraceptive knowledge high	−0.0341	−0.166	−0.0279	0.0126	0.0521
	(0.78)	(1.33)	(0.52)	(0.23)	(1.04)
Mother ≤ 20 years at birth	0.0183	−0.313**	0.0274	0.00381	−0.130**
	(0.49)	(2.96)	(0.60)	(0.08)	(3.03)
Number of household dependents	0.0357	−0.0496	0.00545	0.0147	−0.0411*
(< 18 years)	(1.71)	(1.10)	(0.24)	(0.61)	(2.30)
Post-birth pregnancy interval < 24 months		0.161	−0.0789	−0.00942	−0.0861
		(1.24)	(1.36)	(0.16)	(1.64)
Child health					
Birth weight (kg)		0.510***	−0.0194	−0.0370	−0.0146
		(6.27)	(0.54)	(1.02)	(0.44)
Height (*z*) *t*_1_			0.846***	−0.0341	0.0408
			(67.72)	(1.23)	(1.60)
Height (*z*) *t*_2_				0.0463	−0.000831
				(1.59)	(0.03)
Cognition (*z*) *t*_3_					0.159*** (5.99)
Constant	3.149***	−2.728***	−0.118	−0.356*	0.522***
	(42.85)	(6.14)	(0.70)	(2.11)	(3.63)
Observations	1237	1237	1237	1237	1237

**Table 3:** Linear regression model coefficient estimates (standard errors) among non-first births

	Birthweight (kg)	Height *t*_1_	Height *t*_2_	Cognition *t*_3_	Education *t*_4_

Maternal educational attainment					
Less than primary	0.0239	−0.172	−0.0101	−0.280***	−0.515***
	(0.61)	(1.66)	(0.20)	(5.87)	(9.61)
Primary	−0.0220	−0.0127	−0.00599	−0.111*	−0.237***
	(0.56)	(0.12)	(0.12)	(2.31)	(4.41)
Some secondary	Ref.	Ref.	Ref.	Ref.	Ref.
Upper secondary and above	0.0152	0.227*	0.0123	0.166***	0.152**
	(0.37)	(2.09)	(0.23)	(3.30)	(2.70)
Maternal cognition (*z*)	−0.0128	−0.135**	0.00509	0.0432*	0.00942
	(0.75)	(3.00)	(0.23)	(2.07)	(0.41)
Male	0.0942***	−0.0947	0.00524	−0.0165	−0.189***
	(3.80)	(1.44)	(0.16)	(0.54)	(5.57)
Twins +	−0.408***	−0.348	−0.00452	−0.0647	0.144
	(4.22)	(1.35)	(0.04)	(0.55)	(1.09)
Birth year	0.000574	0.0733***	0.0121*	0.0144**	−0.0291***
	(0.14)	(6.92)	(2.32)	(2.91)	(4.88)
Urban	0.0294	0.191**	0.00585	0.0835*	0.0687
	(1.07)	(2.61)	(0.16)	(2.48)	(1.83)
Maternal marital status	0.0844	−0.181	−0.0231	−0.0580	0.0363
	(1.08)	(0.72)	(0.27)	(0.87)	(0.71)
Fertility characteristics					
Contraceptive knowledge high	0.0619	−0.217*	0.0810	−0.0267	0.0774
	(1.93)	(2.54)	(1.95)	(0.68)	(1.76)
Mother ≤ 20 years at birth	0.143	−0.322	−0.0983	−0.195	−0.0967
	(1.69)	(1.43)	(0.90)	(1.89)	(0.84)
Pre-pregnancy interval < 24 months	−0.0461	−0.312**	−0.0929	−0.103*	−0.0562
	(1.27)	(3.22)	(1.95)	(2.27)	(1.12)
Birth order 3 +	0.0939**	−0.111	−0.0403	−0.0116	−0.0414
	(2.88)	(1.31)	(1.07)	(0.34)	(1.11)
Number of household dependents (< 18 years)	−0.00839	−0.0119	0.0304*	−0.0147	−0.103***
	(0.84)	(0.42)	(2.20)	(1.15)	(7.10)
Post-birth pregnancy interval < 24 months		0.0657	−0.0398	0.00950	−0.106*
		(0.63)	(0.76)	(0.19)	(1.99)
Child Health					
Birth weight (kg)		0.255***	0.0743**	0.0452	0.000836
		(4.72)	(2.82)	(1.80)	(0.03)
Height (*z*) *t*_1_			0.774***	−0.0210	0.0246
			(78.20)	(1.19)	(1.25)
Height (*z*) *t*_2_				0.0225	−0.00459
				(1.16)	(0.21)
Cognition (*z*) *t*_3_					0.240*** (10.55)
Constant	3.046***	−1.905***	−0.717***	−0.331**	0.606***
	(32.97)	(5.76)	(5.25)	(2.73)	(4.95)
Observations	2441	2441	2441	2441	2441

**Table 4:** Maternal fixed effects model

	Birthweight (kg)	Height *t*_1_	Height *t*_2_	Cognition *t*_3_	Education *t*_4_

Male	0.145***	−0.189	−0.0732	0.0104	−0.177***
	(3.94)	(1.85)	(1.34)	(0.24)	(3.76)
Twins +	−0.488*	0.223	−0.583	−0.0163	−0.252
	(2.40)	(0.40)	(1.94)	(0.07)	(0.97)
Birth year	0.00434	0.0867***	0.0241	0.0194	−0.0350***
	(0.42)	(3.51)	(1.94)	(1.74)	(3.35)
Fertility characteristics					
Mother ≤ 20 years at birth	0.0454	−0.294	0.0825	−0.118	−0.276*
	(0.52)	(1.22)	(0.64)	(1.13)	(2.49)
Birth order 1	−0.105	0.173	0.00697	0.0516	0.102
	(1.80)	(1.07)	(0.08)	(0.70)	(1.38)
Birth order 3 +	−0.0190	−0.106	−0.0383	0.0190	−0.0316
	(0.31)	(0.62)	(0.41)	(0.26)	(0.41)
Number of household dependents (< 18 years)	−0.0126	−0.0918	0.0495	0.0177	−0.00403
	(0.49)	(1.16)	(1.27)	(0.47)	(0.12)
Pre-pregnancy interval < 24 months	−0.0496	−0.209	−0.155	−0.0963	−0.0251
	(0.88)	(1.26)	(1.76)	(1.34)	(0.33)
Post-birth pregnancy interval < 24 months		0.169	0.0395	0.00595	−0.0405
		(1.22)	(0.53)	(0.10)	(0.64)
Child health					
Birth weight (kg)		0.294**	0.0460	0.0276	−0.0217
		(3.07)	(0.89)	(0.66)	(0.49)
Height (*z*) *t*_1_			0.684***	0.00189	0.0289
			(36.84)	(0.08)	(1.11)
Height (*z*) *t*_2_				−0.00763	−0.00787
				(0.27)	(0.26)
Cognition (*z*) *t*_3_					0.205***
					(5.53)
Constant	3.209***	−2.132***	−0.740***	−0.531**	0.457**
	(45.30)	(5.51)	(3.67)	(3.01)	(2.77)
Observations	3973	3973	3973	3973	3973

Regression coefficients (with standard errors) based on 3973 births and 3141 mothers

## Data Availability

All data used in the study originated from the RAND Indonesian Family Life Surveys (IFLS), spanning five waves of data collection from 1993 to 2015, by RAND in collaboration with Lembaga Demografi of the University of Indonesia, UCLA, the Population Research center of University of Gadjah Mada, the center for Population and Policy Studies (CPPS) of the University of Gadjah Mada, and Survey METRE. Data collection was supported by the National Institutes of Health. Information on how to obtain the IFLS data files is available on the RAND website (https://www.rand.org/well-being/social-and-behavioral-policy/data/FLS/IFLS/access.html).

## Footnotes

1 The study uses somewhat different categorization of length of birth interval so estimates are not comparable with those derived from studies based on the World Fertility Survey.

2 It is also possible that conditions that precipitate lower fertility (female labor force participation, ideational changes, higher education) sometimes impede breastfeeding Popkin, B. M., Bilsborrow, R. E., & Akin, J. S. (1982). Breast-Feeding Patterns in Low-Income Countries. *Science*, *218*, 1088–1093. http://science.sciencemag.org/content/218/4577/1088.long. Thus, concurrent factors could be countervailing and diminish the net positive effect of fertility decline on human capital accumulation Baum, C. L., & Ford, W. F. (2004). The wage effects of obesity: a longitudinal study. *Health Economics*, *13*(9), 885–899. https://doi.org/10.1002/hec.881.

3 Although there is broad consensus about the child and maternal health benefits of fertility reductions, only a few empirical studies on which the inferences are drawn are anchored in causal analysis—and at least one study conducted under more ideal, quasi-experimental conditions, casts some doubts on the strength of the effects involved Joshi, S., & Schultz, T. P. (2013). Family planning and women’s and children’s health: long-term consequences of an outreach program in Matlab, Bangladesh. *Demography*, *50*(1), 149–180. https://doi.org/10.1007/s13524-012-0172-2.
