# Supplementary material for: The reach of fertility decline: a longitudinal analysis of human capital gains across generations
Source: Genus. Author manuscript; Available in PMC 2026 Nov 7. (PMC13742192; doi:10.1186/s41118-022-00176-4)
Supplement: Supplementary file 1 [file NIHMS2173790-supplement-Supplementary_Material.docx]

ONLINE SUPPLEMENTARY MATERIALS

[Figure S1. Indonesia Fertility Transition	2](#_Toc109218926)

[Table S1: IFLS birth cohort sample	3](#_Toc109218927)

[Table S2: Baseline model set estimation equations and terms	4](#_Toc109218928)

[Table S3: Fertility characteristics by maternal education	6](#_Toc109218929)

[Table S4: Fertility and offspring education: Accounting for child health	7](#_Toc109218930)

[Table S5: Counterfactual scenario mean and distributional shifts	8](#_Toc109218931)

[Figure S2. Simulated counterfactual scenarios by birth order	9](#_Toc109218932)

[Table S6: Sensitivity Check: Adjusting for sibling survivorship and pregnancy losses	10](#_Toc109218933)

[Table S7: Maternal fixed effects models of fertility and offspring education: Accounting for child health	13](#_Toc109218934)

[Table S8: Maternal Fixed Effects Simulation Results	14](#_Toc109218935)

[Table S9: Fertility, child health, and offspring education: Breastfeeding role	15](#_Toc109218936)

Figure S1. Indonesia Fertility Transition. Province-level total fertility rate (TFR) between 1990 and 2000.

|  |  |  |  |  |  |  |  |  |  |  |  |  |  |  |  |  |  |  |  |  |
| --- | --- | --- | --- | --- | --- | --- | --- | --- | --- | --- | --- | --- | --- | --- | --- | --- | --- | --- | --- | --- |
| 1990 | 4.37 | 4.29 | 3.89 | 4.09 | 3.76 | 4.22 | 3.97 | 4.05 | 2.33 | 3.47 | 3.05 | 2.08 | 2.46 | 2.28 | 4.98 | 4.61 | 4.44 | 4.03 | 3.24 | 3.28 |
| 1994 | 3.3 | 3.88 | 3.19 | 3.1 | 2.97 | 2.87 | 3.45 | 3.45 | 1.9 | 3.17 | 2.77 | 1.79 | 2.22 | 2.14 | 3.64 | 3.87 | 3.34 | 2.31 | 2.33 | 3.21 |
| 1997 | 2.81 | 3.1 | 3.06 | 2.77 | 2.67 | 2.88 | 2.68 | 2.65 | 1.63 | 2.51 | 2.06 | 1.44 | 1.71 | 1.89 | 2.92 | 3.37 | 2.99 | 2.74 | 2.33 | 2.5 |
| 2000 | 2.44 | 2.84 | 2.95 | 2.45 | 2.37 | 2.33 | 2.49 | 2.42 | 1.66 | 2.28 | 2.14 | 1.79 | 1.87 | 2.03 | 2.69 | 3.46 | 2.62 | 2.21 | 2.3 | 2.32 |

Source: Data from Badan Pusat Statistik (BPS) n/d and Hull 2016.

Note: Each line displays the TFR trajectory for a single province.

Table S1: IFLS birth cohort sample. Descriptive statistics for all eligible IFLS births (between 1990 and 2000), including individuals with incomplete data or lost to follow-up (n=6,627).

|  | **Mean or Percent** | **Total non-missing** |
| --- | --- | --- |
| **Maternal characteristics** |  |  |
| Maternal highest education level |  |  |
| Less than primary | 24.2% | 2,623 |
| Primary | 27.7% | 2,623 |
| Some secondary | 20.2% | 2,623 |
| Upper secondary school and above | 27.9% | 2,623 |
| Maternal cognition (z) | 0.147 | 2,418 |
| Contraceptive knowledge high | 77.6% | 2,858 |
| Total live births |  |  |
| 1 | 21.6% | 2,858 |
| 2 | 36.5% | 2,858 |
| 3 | 20.1% | 2,858 |
| ≥4 | 21.8% | 2,858 |
| Mother married at birth | 88.8% | 2,818 |
| **Birth characteristics** |  |  |
| Urban | 44.9% | 6,625 |
| Male | 51.4% | 6,627 |
| *Fertility characteristics* |  |  |
| Mother ≤20 years-old at birth | 12.6% | 6,626 |
| Multiple gestation birth | 1.7% | 6,627 |
| Birth order |  |  |
| 1 | 33.6% | 6,627 |
| 2 | 24.9% | 6,627 |
| 3+ | 41.5% | 6,627 |
| Number of children (<18 years-old) in household at birth | 2.960 | 4,227 |
| Pre-pregnancy interval <24 months | 9.7% | 6,599 |
| Post-birth pregnancy interval <24 months | 13.8% | 6,462 |
| *Child health characteristics* |  |  |
| Birth weight (kg) | 3.202 | 6,010 |
| Height (z) t_1_ | -1.470 | 5,410 |
| Height (z) t_2_ | -1.545 | 5,652 |
| Cognition (z) t_3_ | -0.428 | 5,972 |
| Years of educational attainment (2014) | 9.936 | 5,880 |
| *Source: Indonesian Family Life Survey Waves 1-5* |  |  |

Table S2: Baseline model set estimation equations and terms. Each equation below relates outcomes to fertility and child health predictors at the index child level (i), with fixed effects to account for unobserved time-invariant heterogeneities across province (j), and measurements taken longitudinally over time (t).

| **B**_ij(t=0)_ | = **α**_0j_ + **α**_v_ **V**_ij(t=0)_ + *e*_Bij_ | (1) |
| --- | --- | --- |
| **S**_ij(t=1)_ | = **β**_0j_ + **β**_w_ **W**_ij(t=1)_+ *e*s_1ij_ | (2) |
| **S**_ij(t=2)_ | = **γ**_0j_ + **γ**_x_ **X** _ij(t=2)_ + *e*s_2ij_ | (3) |
| **C**_ij(t=3)_ | = **δ**_0j_ + **δ**_y_ **Y**_ij(t=3)_ + *e*_Cij_ | (4) |
| **E**_ij(t=4)_ | = **ζ**_0j_ + **ζ**_z_ **Z**_ij(t=4)_ + *e*_Eij_ | (5) |

| **Equation** | **Terms** |
| --- | --- |
| **1** | **B**_ij_: Birthweight (kilograms)  **V**_ij(t=0)_: Characteristics defined before birth, including maternal fertility measures (high contraceptive knowledge; maternal age ≤ 20 years old at birth; first birth; birth order ≥ 3; number of children in household at birth; pre-pregnancy interval <24 months), maternal education (categorized as less than primary complete, primary complete, some secondary complete, and upper secondary school or above complete), and covariates (maternal cognition z-score; mother married at birth; urban residence; multiple gestation birth; and infant sex male).  **α**_0j_: Province-specific birth weight intercept.  **α**_v_: Vector of coefficients predicting birth weight, aligned with **V**_ij(t=0)_.  ***e***_Bij_: Idiosyncratic errors in birth weight. |
| **2** | **S**_ij(t=1)_: Physical growth (height-for-age z-score) at time *t*.  **W**_ij(t=1)_: Outcome estimate from preceding model (**B**_ij_), maternal fertility measures (high contraceptive knowledge; maternal age ≤ 20 years old at birth; first birth; birth order ≥ 3; number of children in household at time *t*; pre-pregnancy interval <24 months; post-birth pregnancy interval < 24 months), maternal education (categorized as less than primary complete, primary complete, some secondary complete, and upper secondary school or above complete), and covariates (maternal cognition z-score; mother marital status at time *t*; urban residence; multiple gestation birth; and infant sex male).  **β** _0j_: Province-specific height-for-age intercept at time *t*.  **Β**_w_: Vector of coefficients predicting height-for-age, aligned with **W**_ij(t=1)_.  ***e***_S1ij_: Idiosyncratic errors in height-for-age at time *t*. |
| **3** | **S**_ij(t=2)_: Physical growth (height-for-age z-score) at time *t*.  **X**_ij(t=2)_: Outcome estimate from preceding model (**B**_ij_, **S**_ij(t=1)_), maternal fertility measures (high contraceptive knowledge; maternal age ≤ 20 years old at birth; first birth; birth order ≥ 3; number of children in household at time *t*; pre-pregnancy interval <24 months; post-birth pregnancy interval < 24 months), maternal education (categorized as less than primary complete, primary complete, some secondary complete, and upper secondary school or above complete), and covariates (maternal cognition z-score; mother marital status at *t*; urban residence; multiple gestation birth; and infant sex male).  **γ** _0j_: Province-specific height-for-age intercept at time *t*.  **γ**_x_: Vector of coefficients predicting height-for-age, aligned with **X**_ij(t=2)_.  ***e***_S2ij_: Idiosyncratic errors in height-for-age at time *t*. |
| **4** | **C**_ij_: Cognitive ability (age-standardized cognitive test score).  **Y**_ij(t=3)_: Outcome estimate from preceding model (**B**_ij_, **S**_ij(t=1)_, **S**_ij(t=2)_), maternal fertility measures (high contraceptive knowledge; maternal age ≤ 20 years old at birth; first birth; birth order ≥ 3; number of children in household at time *t*; pre-pregnancy interval <24 months; post-birth pregnancy interval < 24 months), maternal education (categorized as less than primary complete, primary complete, some secondary complete, and upper secondary school or above complete), and covariates (maternal cognition z-score; mother marital status at *t*; urban residence; multiple gestation birth; and infant sex male).  **δ**_0j_: Province-specific age-standardized cognitive test score intercept at time *t*.  **δ**_y_: Vector of coefficients predicting age-standardized cognitive test score, aligned with **Y**_ij(t=3)_.  ***e***_Cij_: Idiosyncratic errors in age-standardized cognitive test score at time *t*. |
| **5** | **E**_ij_: Educational attainment (age-standardized years of schooling).  **Z**_ij(t=4)_: Outcome estimate from preceding model (**B**_ij_, **S**_ij(t=1)_, **S**_ij(t=2)_, **C**_ij_), maternal fertility measures (high contraceptive knowledge; maternal age ≤ 20 years old at birth; first birth; birth order ≥ 3; number of children in household at time *t*; pre-pregnancy interval <24 months; post-birth pregnancy interval < 24 months), maternal education (categorized as less than primary complete, primary complete, some secondary complete, and upper secondary school or above complete), and covariates (maternal cognition z-score; mother marital status at *t*; urban residence; multiple gestation birth; and infant sex male).  **ζ**_0j_: Province-specific age-standardized years of schooling intercept at time *t*.  **ζ**_z_: Vector of coefficients predicting age-standardized years of schooling, aligned with **Z**_ij(t=4)_.  ***e***_Eij_: Idiosyncratic errors in age-standardized years of schooling at time *t*. |

Table S3: Fertility characteristics by maternal education. Fertility-related factors summarized by maternal education subgroups, based on complete cases.

| (a) First births | Less than primary | Primary complete | Some secondary school | Upper secondary school and above |
| --- | --- | --- | --- | --- |
| Knowledge of contraceptives¹ | 64.1% | 69.6% | 84.8% | 88.8% |
| Mother's age at childbirth ≤20 | 40.5% | 41.0% | 39.5% | 10.8% |
| Post-birth pregnancy interval <24 months | 21.4% | 12.3% | 13.4% | 20.0% |
| Mean number of children in household |  |  |  |  |
| At birth | 1.31 | 1.34 | 1.29 | 1.32 |
| t_1_ | 1.71 | 1.67 | 1.66 | 1.60 |
| t_2_ | 2.07 | 1.94 | 1.91 | 2.04 |
| t_3_ | 2.31 | 2.17 | 2.10 | 2.27 |
| t_4_ | 1.95 | 1.80 | 1.92 | 1.97 |

| (b) Non-first births | Less than primary | Primary complete | Some secondary school | Upper secondary school and above |
| --- | --- | --- | --- | --- |
| Knowledge of contraceptives¹ | 62.6% | 75.1% | 87.9% | 92.1% |
| Mother's age at childbirth ≤20 | 1.5% | 5.0% | 1.6% | 0.9% |
| Birth order ≥ 3 | 79.8% | 59.4% | 53.6% | 39.8% |
| Pre-pregnancy interval <24 months | 13.7% | 12.8% | 16.6% | 17.1% |
| Post-birth pregnancy interval <24 months | 12.4% | 11.3% | 13.3% | 9.7% |
| Mean number of children in household |  |  |  |  |
| At birth | 3.79 | 3.15 | 3.10 | 2.86 |
| t_1_ | 3.58 | 3.10 | 3.02 | 2.97 |
| t_2_ | 3.37 | 3.07 | 3.10 | 3.01 |
| t_3_ | 2.96 | 2.82 | 2.94 | 2.90 |
| t_4_ | 1.44 | 1.52 | 1.62 | 1.46 |

¹ Based on whether mother reported knowing about oral contraceptives, injections, IUDs, and Norplant

Table S4: Fertility and offspring education: Accounting for child health. Staged regressions of education z-score on (1) maternal education and fertility, and (2) with child health measures added. Both models adjusted for maternal cognition, maternal marital status, infant sex, urbanicity, multiple births, and birth year.

|  | First births | | Non-first births | |
| --- | --- | --- | --- | --- |
|  | (1) | (2) | (3) | (4) |
| **Maternal education completed** |  |  |  |  |
| Less than primary | -0.485*** | -0.417*** | -0.584*** | -0.515*** |
|  | (-6.75) | (-5.83) | (-10.74) | (-9.61) |
| Primary school | -0.263*** | -0.233*** | -0.264*** | -0.237*** |
|  | (-4.92) | (-4.40) | (-4.82) | (-4.41) |
| Some secondary | *REF* | *REF* | *REF* | *REF* |
| Upper secondary school and above | 0.205*** | 0.176*** | 0.194*** | 0.152** |
|  | (4.12) | (3.59) | (3.40) | (2.70) |
| **Fertility-related characteristics** |  |  |  |  |
| Contraceptive knowledge high^1^ | 0.0468 | 0.0521 | 0.0684 | 0.0774 |
|  | (0.91) | (1.04) | (1.53) | (1.76) |
| Maternal age at birth ≤20 years-old | -0.142** | -0.130** | -0.146 | -0.0967 |
|  | (-3.26) | (-3.03) | (-1.24) | (-0.84) |
| Pre-pregnancy interval <24 months | - | - | -0.0882 | -0.0562 |
|  |  |  | (-1.73) | (-1.12) |
| *Birth order* |  |  |  |  |
| 1 | - | - | - | - |
| 2 | *-* | *-* | *REF* | *REF* |
| 3+ | *-* | *-* | -0.0469 | -0.0414 |
|  |  |  | (-1.23) | (-1.11) |
| Post-birth pregnancy interval <24 months | -0.0785 | -0.0861 | -0.105 | -0.106* |
|  | (-1.47) | (-1.64) | (-1.92) | (-1.99) |
| Number of children in household (t_4_) | -0.0457* | -0.0411* | -0.111*** | -0.103*** |
|  | (-2.52) | (-2.30) | (-7.50) | (-7.10) |
| **Birth & child characteristics** |  |  |  |  |
| Birth weight (kg) | - | -0.0146 | - | 0.000836 |
|  |  | (-0.44) |  | (0.03) |
| Height-for-age t_1_ | - | 0.0408 | - | 0.0246 |
|  |  | (1.60) |  | (1.25) |
| Height-for-age t_2_ | - | -0.000831 | - | -0.00459 |
|  |  | (-0.03) |  | (-0.21) |
| Cognition-for-age t_3_ | - | 0.159*** | - | 0.240*** |
|  |  | (5.99) |  | (10.55) |

Standard errors in parentheses. ^†^p<0.1 * p<0.05 ** p<0.01 *** p<0.001

¹ Based on whether mother reported knowing about oral contraceptives, injections, IUDs, and Norplant

Table S5: Counterfactual scenario mean and distributional shifts. Outcome distribution summary measures for each child health and educational outcome modeled under following scenarios: (A) baseline (no manipulation); (B) exogenous change in family planning services (low fertility regime); (C) exogenous shift to high maternal education (including intermediate fertility shifts); and (D) scenarios (B) and (C) combined.

|  | Birth weight | Height (t1) | | Height (t2) | | Cognition (t3) | | Educational attainment | |
| --- | --- | --- | --- | --- | --- | --- | --- | --- | --- |
|  | kg | z-score | cm | z-score | cm | z-score | score | z-score | years |
| First births | |  |  |  |  |  |  |  |  |
| A | 3.10 | -1.36 | 75.66 | -1.39 | 109.34 | -0.31 | 11.21 | 0.28 | 11.15 |
| B | 3.08 | -1.31 | 75.66 | -1.35 | 109.34 | -0.32 | 11.16 | 0.39 | 11.39 |
| C | 3.10 | -1.13 | 75.66 | -1.13 | 109.35 | -0.15 | 11.71 | 0.55 | 11.77 |
| D | 3.09 | -1.13 | 75.67 | -1.12 | 109.35 | -0.17 | 11.66 | 0.63 | 11.94 |
| Non-first births | |  |  |  |  |  |  |  |  |
| A | 3.26 | -1.55 | 75.65 | -1.58 | 109.33 | -0.39 | 10.96 | 0.08 | 10.68 |
| B | 3.23 | -1.48 | 75.65 | -1.50 | 109.34 | -0.35 | 11.07 | 0.21 | 10.98 |
| C | 3.26 | -1.32 | 75.66 | -1.37 | 109.34 | -0.13 | 11.80 | 0.54 | 11.75 |
| D | 3.24 | -1.23 | 75.66 | -1.29 | 109.34 | -0.09 | 11.90 | 0.64 | 11.98 |
| Aggregated | |  |  |  |  |  |  |  |  |
| A | 3.21 | -1.49 | 75.65 | -1.52 | 109.33 | -0.36 | 11.05 | 0.15 | 10.84 |
| B | 3.16 | -1.40 | 75.66 | -1.42 | 109.34 | -0.34 | 11.12 | 0.30 | 11.18 |
| C | 3.19 | -1.23 | 75.66 | -1.25 | 109.35 | -0.14 | 11.76 | 0.55 | 11.76 |
| D | 3.16 | -1.18 | 75.66 | -1.21 | 109.35 | -0.13 | 11.78 | 0.63 | 11.96 |

Figure S2. Simulated counterfactual scenarios by birth order**:** (A) First birth (FB) baseline (no manipulation); (B) FB exogenous change in family planning services (low fertility regime); (C) FB exogenous shift in maternal education (including intermediate fertility shifts); (D) FB scenarios (B) and (C) combined; (E) Non-FB (NFB) baseline (no manipulation); (F) NFB exogenous change in family planning services (low fertility regime); (G) NFB exogenous shift in maternal education (including intermediate fertility shifts); and (H) NFB scenarios (F) and (G) combined.

Table S6: Sensitivity Check: Adjusting for sibling survivorship and pregnancy losses. Regression coefficients (with standard errors), with additional adjustments for a prior pregnancy or child loss prior to each respective outcome among (a) first births and (b) non-first births.

| (a) First births | **Birthweight (kg)** | **Height**  **t_1_** | **Height t_2_** | **Cognition t_3_** | **Education t_4_** |
| --- | --- | --- | --- | --- | --- |
| *Maternal educational attainment* |  |  |  |  |  |
| Less than primary | -0.0874 | -0.0706 | -0.0835 | -0.413*** | -0.418*** |
|  | (-1.42) | (-0.40) | (-1.10) | (-5.39) | (-5.84) |
| Primary | -0.0695 | -0.0219 | -0.0994 | -0.179** | -0.235*** |
|  | (-1.51) | (-0.17) | (-1.76) | (-3.13) | (-4.44) |
| Some secondary | REF | REF | REF | REF | REF |
| Upper secondary school and above | -0.0321 | 0.272* | 0.0672 | 0.101 | 0.177*** |
|  | (-0.75) | (2.26) | (1.28) | (1.90) | (3.60) |
| Maternal cognition (z) | 0.00661 | -0.0106 | 0.00706 | 0.0627* | 0.0637* |
|  | (0.27) | (-0.16) | (0.24) | (2.09) | (2.29) |
| Male | 0.0215 | -0.0574 | 0.0130 | -0.0460 | -0.116** |
|  | (0.67) | (-0.63) | (0.33) | (-1.16) | (-3.16) |
| Twins + | -0.454** | -0.831* | -0.0302 | 0.164 | 0.113 |
|  | (-3.20) | (-2.05) | (-0.17) | (0.92) | (0.69) |
| Birth year | -0.00392 | 0.0659*** | 0.0156* | -0.000963 | -0.0245*** |
|  | (-0.66) | (4.35) | (2.35) | (-0.14) | (-3.57) |
| Urban | -0.0277 | 0.0982 | 0.0490 | 0.104* | -0.00846 |
|  | (-0.77) | (0.97) | (1.11) | (2.34) | (-0.21) |
| Maternal marital status | -0.0255 | -0.142 | -0.0934 | 0.150 | 0.131 |
|  | (-0.59) | (-0.44) | (-0.93) | (1.66) | (1.84) |
| *Fertility characteristics* |  |  |  |  |  |
| Contraceptive knowledge high | -0.0341 | -0.166 | -0.0279 | 0.0131 | 0.0516 |
|  | (-0.78) | (-1.34) | (-0.52) | (0.24) | (1.03) |
| Mother ≤20 years-old at birth | 0.0206 | -0.311** | 0.0279 | -0.00275 | -0.124** |
|  | (0.55) | (-2.92) | (0.60) | (-0.06) | (-2.88) |
| Number of household dependents (<18 years-old) | 0.0356 | -0.0497 | 0.00535 | 0.0167 | -0.0407* |
|  | (1.70) | (-1.10) | (0.23) | (0.69) | (-2.28) |
| Post-birth pregnancy interval <24 months |  | 0.159 | -0.0792 | -0.00575 | -0.0908 |
|  |  | (1.23) | (-1.37) | (-0.10) | (-1.73) |
| *Child health* |  |  |  |  |  |
| Birth weight (kg) |  | 0.510*** | -0.0194 | -0.0374 | -0.0144 |
|  |  | (6.27) | (-0.54) | (-1.03) | (-0.43) |
| Height (z) t1 |  |  | 0.846*** | -0.0341 | 0.0409 |
|  |  |  | (67.69) | (-1.23) | (1.60) |
| Height (z) t2 |  |  |  | 0.0465 | -0.00109 |
|  |  |  |  | (1.60) | (-0.04) |
| Cognition (z) t3 |  |  |  |  | 0.160*** |
|  |  |  |  |  | (6.05) |
| Prior fetal or child death | 0.0393 | 0.0412 | 0.00777 | -0.103 | 0.0976 |
|  | (0.60) | (0.26) | (0.11) | (-1.47) | (1.52) |
| Constant | 3.147*** | -2.728*** | -0.119 | -0.345* | 0.512*** |
|  | (42.77) | (-6.14) | (-0.70) | (-2.05) | (3.55) |
| Observations | 1237 | 1237 | 1237 | 1237 | 1237 |

| (b) Non-first births | **Birthweight (kg)** | **Height t_1_** | **Height t_2_** | **Cognition t_3_** | **Education t_4_** |
| --- | --- | --- | --- | --- | --- |
| *Maternal educational attainment* |  |  |  |  |  |
| Less than primary | 0.0233 | -0.176 | -0.0104 | -0.279*** | -0.514*** |
|  | (0.60) | (-1.70) | (-0.21) | (-5.85) | (-9.60) |
| Primary | -0.0215 | -0.0113 | -0.00571 | -0.112* | -0.237*** |
|  | (-0.55) | (-0.11) | (-0.11) | (-2.33) | (-4.42) |
| Some secondary | REF | REF | REF | REF | REF |
| Upper secondary school and above | 0.0165 | 0.232* | 0.0130 | 0.163** | 0.150** |
|  | (0.40) | (2.13) | (0.25) | (3.25) | (2.68) |
| Maternal cognition (z) | -0.0128 | -0.135** | 0.00505 | 0.0433* | 0.00959 |
|  | (-0.75) | (-2.99) | (0.23) | (2.08) | (0.41) |
| Male | 0.0947*** | -0.0904 | 0.00575 | -0.0185 | -0.190*** |
|  | (3.82) | (-1.37) | (0.18) | (-0.61) | (-5.59) |
| Twins + | -0.406*** | -0.350 | -0.00432 | -0.0659 | 0.143 |
|  | (-4.20) | (-1.36) | (-0.03) | (-0.56) | (1.08) |
| Birth year | 0.00124 | 0.0768*** | 0.0126* | 0.0126* | -0.0301*** |
|  | (0.30) | (7.07) | (2.34) | (2.49) | (-4.91) |
| Urban | 0.0282 | 0.184* | 0.00509 | 0.0865* | 0.0703 |
|  | (1.02) | (2.51) | (0.14) | (2.56) | (1.86) |
| Maternal marital status | 0.0859 | -0.172 | -0.0236 | -0.0553 | 0.0369 |
|  | (1.10) | (-0.69) | (-0.28) | (-0.83) | (0.72) |
| *Fertility characteristics* |  |  |  |  |  |
| Contraceptive knowledge high | 0.0614 | -0.218* | 0.0809 | -0.0260 | 0.0779 |
|  | (1.91) | (-2.56) | (1.95) | (-0.66) | (1.77) |
| Mother ≤20 years-old at birth | 0.141 | -0.341 | -0.101 | -0.185 | -0.0916 |
|  | (1.66) | (-1.52) | (-0.92) | (-1.79) | (-0.79) |
| Pre-pregnancy interval <24 months | -0.0485 | -0.324*** | -0.0946* | -0.0969* | -0.0537 |
|  | (-1.33) | (-3.34) | (-1.98) | (-2.12) | (-1.07) |
| Birth order 3+ | 0.0911** | -0.129 | -0.0420 | -0.00631 | -0.0389 |
|  | (2.78) | (-1.52) | (-1.11) | (-0.18) | (-1.03) |
| Number of household dependents (<18 years-old) | -0.00778 | -0.00596 | 0.0309* | -0.0158 | -0.102*** |
|  | (-0.78) | (-0.21) | (2.23) | (-1.23) | (-7.03) |
| Post-birth pregnancy interval <24 months |  | 0.0533 | -0.0415 | 0.0151 | -0.104 |
|  |  | (0.51) | (-0.79) | (0.30) | (-1.95) |
| *Child Health* |  |  |  |  |  |
| Birth weight (kg) |  | 0.253*** | 0.0741** | 0.0460 | 0.00126 |
|  |  | (4.69) | (2.81) | (1.84) | (0.05) |
| Height (z) t1 |  |  | 0.774*** | -0.0207 | 0.0248 |
|  |  |  | (78.14) | (-1.17) | (1.26) |
| Height (z) t2 |  |  |  | 0.0226 | -0.00455 |
|  |  |  |  | (1.17) | (-0.21) |
| Cognition (z) t3 |  |  |  |  | 0.239*** |
|  |  |  |  |  | (10.53) |
| Prior fetal or child death | 0.0264 | 0.124 | 0.0156 | -0.0615 | -0.0312 |
|  | (0.80) | (1.46) | (0.38) | (-1.58) | (-0.72) |
| Constant | 3.040*** | -1.938*** | -0.720*** | -0.324** | 0.608*** |
|  | (32.79) | (-5.85) | (-5.26) | (-2.66) | (4.97) |
| Observations | 2441 | 2441 | 2441 | 2441 | 2441 |

Table S7: Maternal fixed effects models of fertility and offspring education: Accounting for child health. Staged regressions of education z-score on (1) maternal education and fertility, and (2) with child health measures added. Both models adjust for male sex,

|  | (1) | (2) |
| --- | --- | --- |
| **Fertility-related characteristics** |  |  |
| Maternal age at birth ≤20 years-old | -0.311** | -0.276* |
|  | (-2.76) | (-2.49) |
| Pre-pregnancy interval <24 months | -0.0480 | -0.0251 |
|  | (-0.62) | (-0.33) |
| *Birth order* |  |  |
| 1 | 0.115 | 0.102 |
|  | (1.53) | (1.38) |
| 2 | *REF* | *REF* |
| 3+ | -0.0272 | -0.0316 |
|  | (-0.35) | (-0.41) |
| Post-birth pregnancy interval <24 months | -0.0344 | -0.0405 |
|  | (-0.53) | (-0.64) |
| Number of children in household (t_4_) | -0.0134 | -0.00403 |
|  | (-0.41) | (-0.12) |
| **Birth & child characteristics** |  |  |
| Birth weight (kg) |  | -0.0217 |
|  |  | (-0.49) |
| Height-for-age t_1_ |  | 0.0289 |
|  |  | (1.11) |
| Height-for-age t_2_ |  | -0.00787 |
|  |  | (-0.26) |
| Cognition-for-age t_3_ |  | 0.205*** |
|  |  | (5.53) |

Standard errors in parentheses. ^†^p<0.1 * p<0.05 ** p<0.01 *** p<0.001

¹ Based on whether mother reported knowing about oral contraceptives, injections, IUDs, and Norplant

Table S8: Maternal Fixed Effects Simulation Results. Outcome distribution summary measures for baseline and low fertility scenario simulations.

|  | Birth weight (kg) | | Height-for-age z (t1) | | Height-for-age z (t2) | | Cognition-for-age z (t3) | | Education-for-age z (t4) | | |  |
| --- | --- | --- | --- | --- | --- | --- | --- | --- | --- | --- | --- | --- |
|  | Baseline | Low fertility | Baseline | Low fertility | Baseline | Low fertility | Baseline | Low fertility | | Baseline | Low fertility | |
| Global mean | 3.21 | 3.20 | -1.76 | -1.54 | -2.09 | -1.80 | -0.67 | -0.58 | | 0.04 | 0.11 | |
| Maternal minimum | 2.95 | 2.95 | -2.48 | -2.25 | -2.73 | -2.42 | -0.97 | -0.89 | | -0.29 | -0.22 | |
| First birth value | 3.17 | 3.17 | -1.39 | -1.20 | -1.67 | -1.42 | -0.62 | -0.47 | | 0.07 | 0.21 | |
| Second birth value | 3.25 | 3.25 | -1.67 | -1.68 | -1.99 | -1.99 | -0.69 | -0.69 | | 0.03 | 0.03 | |

Table S9: Fertility, child health, and offspring education: Breastfeeding role. Model set rerun to assess how incorporating breastfeeding changes model coefficients for child health and education.

| (a) First births | **Breastfeeding (months)** | **Height**  **t_1_** | **Height t_2_** | | **Cognition t_3_** | **Education t_4_** |
| --- | --- | --- | --- | --- | --- | --- |
| *Maternal educational attainment* |  |  | |  |  |  |
| Less than primary | 1.298 | -0.0370 | | -0.0804 | -0.410*** | -0.418*** |
|  | (1.42) | (-0.22) | | (-1.09) | (-5.40) | (-5.77) |
| Primary | 0.0371 | -0.0377 | | -0.107 | -0.175** | -0.236*** |
|  | (0.05) | (-0.30) | | (-1.94) | (-3.07) | (-4.40) |
| Some secondary | REF | REF | | REF | REF | REF |
| Upper secondary school and above | -0.0870 | 0.230 | | 0.0725 | 0.121* | 0.172*** |
|  | (-0.14) | (1.93) | | (1.41) | (2.27) | (3.44) |
| Maternal cognition (z) | -0.345 | -0.0275 | | 0.00776 | 0.0650* | 0.0649* |
|  | (-0.96) | (-0.41) | | (0.27) | (2.17) | (2.30) |
| Male | -0.138 | -0.0347 | | 0.0243 | -0.0449 | -0.118** |
|  | (-0.29) | (-0.39) | | (0.63) | (-1.13) | (-3.16) |
| Twins + | 3.504 | -0.666 | | -0.0192 | 0.207 | 0.134 |
|  | (1.61) | (-1.64) | | (-0.11) | (1.14) | (0.79) |
| Birth year | -1.300*** | 0.0130 | | 0.0107 | 0.00314 | -0.0256*** |
|  | (-14.63) | (0.79) | | (1.51) | (0.43) | (-3.40) |
| Urban | -0.590 | 0.0798 | | 0.0258 | 0.100* | -0.00758 |
|  | (-1.10) | (0.80) | | (0.60) | (2.26) | (-0.18) |
| Maternal marital status | 0.953 | -0.210 | | -0.0937 | 0.161 | 0.140 |
|  | (1.48) | (-0.67) | | (-0.97) | (1.80) | (1.94) |
| *Fertility characteristics* |  |  | |  |  |  |
| Contraceptive knowledge high | 1.337* | -0.128 | | -0.00960 | 0.0174 | 0.0540 |
|  | (2.05) | (-1.05) | | (-0.18) | (0.32) | (1.06) |
| Mother ≤20 years-old at birth | 0.527 | -0.297** | | 0.0301 | -0.00503 | -0.126** |
|  | (0.94) | (-2.86) | | (0.67) | (-0.11) | (-2.88) |
| Number of household dependents (<18 years-old) | -1.555*** | -0.0939* | | 0.0231 | 0.0137 | -0.0413* |
|  | (-5.01) | (-2.12) | | (1.04) | (0.57) | (-2.28) |
| Post-birth pregnancy interval <24 months | -2.401*** | 0.0663 | | -0.0950 | -0.00191 | -0.0834 |
|  | (-3.55) | (0.52) | | (-1.67) | (-0.03) | (-1.56) |
| *Child health* |  |  | |  |  |  |
| Birth weight (kg) | -0.350 | 0.493*** | | -0.0211 | -0.0385 | -0.0148 |
|  | (-0.81) | (6.16) | | (-0.60) | (-1.06) | (-0.43) |
| Height (z) t1 |  |  | | 0.849*** | -0.0278 | 0.0374 |
|  |  |  | | (67.64) | (-0.97) | (1.39) |
| Height (z) t2 |  |  | |  | 0.0439 | 0.00134 |
|  |  |  | |  | (1.46) | (0.05) |
| Cognition (z) t3 |  |  | |  |  | 0.161*** |
|  |  |  | |  |  | (5.88) |
| Prior fetal or child death | -1.775 | 0.0340 | | -0.0214 | -0.0546 | 0.0914 |
|  | (-1.81) | (0.22) | | (-0.32) | (-0.78) | (1.40) |
| Breastfeeding |  | -0.0440*** | | -0.00342 | 0.00200 | -0.00120 |
|  |  | (-8.19) | | (-1.44) | (0.82) | (-0.52) |
| Constant | 18.29*** | -1.832*** | | -0.0951 | -0.385* | 0.522*** |
|  | (10.50) | (-4.10) | | (-0.57) | (-2.26) | (3.50) |
| Observations | 1212 | 1212 | 1212 | | 1212 | 1212 |

| (b) Non-first births | **Breastfeeding (months)** | **Height**  **t_1_** | **Height t_2_** | | **Cognition t_3_** | **Education t_4_** |
| --- | --- | --- | --- | --- | --- | --- |
| *Maternal educational attainment* |  |  | |  |  |  |
| Less than primary | 0.906 | -0.137 | | -0.0112 | -0.273*** | -0.511*** |
|  | (1.65) | (-1.35) | | (-0.22) | (-5.70) | (-9.50) |
| Primary | 0.166 | -0.0102 | | -0.00598 | -0.109* | -0.230*** |
|  | (0.30) | (-0.10) | | (-0.12) | (-2.26) | (-4.28) |
| Some secondary | REF | REF | | REF | REF | REF |
| Upper secondary school and above | -0.354 | 0.215* | | 0.0138 | 0.166** | 0.156** |
|  | (-0.62) | (2.02) | | (0.26) | (3.29) | (2.77) |
| Maternal cognition (z) | -0.396 | -0.149*** | | 0.00660 | 0.0467* | 0.00756 |
|  | (-1.65) | (-3.37) | | (0.30) | (2.22) | (0.32) |
| Male | -0.107 | -0.0904 | | 0.0103 | -0.0164 | -0.191*** |
|  | (-0.31) | (-1.40) | | (0.32) | (-0.54) | (-5.61) |
| Twins + | -1.880 | -0.404 | | -0.00394 | -0.0599 | 0.127 |
|  | (-1.39) | (-1.61) | | (-0.03) | (-0.51) | (0.97) |
| Birth year | -1.134*** | 0.0352** | | 0.0145* | 0.0182*** | -0.0339*** |
|  | (-19.64) | (3.07) | | (2.51) | (3.33) | (-5.21) |
| Urban | -1.181** | 0.134 | | 0.00286 | 0.0905** | 0.0657 |
|  | (-3.06) | (1.87) | | (0.08) | (2.67) | (1.74) |
| Maternal marital status | 1.552 | -0.230 | | -0.0220 | -0.0453 | 0.0346 |
|  | (1.42) | (-0.95) | | (-0.26) | (-0.67) | (0.68) |
| *Fertility characteristics* |  |  | |  |  |  |
| Contraceptive knowledge high | 0.818 | -0.205* | | 0.0791 | -0.0271 | 0.0812 |
|  | (1.81) | (-2.45) | | (1.89) | (-0.69) | (1.84) |
| Mother ≤20 years-old at birth | -0.346 | -0.379 | | -0.109 | -0.198 | -0.0911 |
|  | (-0.29) | (-1.72) | | (-0.99) | (-1.90) | (-0.78) |
| Number of household dependents (<18 years-old) | 0.113 | -0.0123 | | 0.0321* | -0.0132 | -0.106*** |
|  | (0.80) | (-0.44) | | (2.29) | (-1.03) | (-7.26) |
| Birth order 3+ | 0.239 | -0.116 | | -0.0410 | -0.0105 | -0.0336 |
|  | (0.52) | (-1.38) | | (-1.08) | (-0.31) | (-0.89) |
| Pre-pregnancy interval <24 months | 0.415 | -0.324*** | | -0.0932 | -0.102* | -0.0530 |
|  | (0.81) | (-3.41) | | (-1.93) | (-2.23) | (-1.05) |
| Post-birth pregnancy interval <24 months | -3.789*** | -0.103 | | -0.0282 | 0.0270 | -0.113* |
|  | (-6.90) | (-1.00) | | (-0.53) | (0.54) | (-2.10) |
| *Child health* |  |  | |  |  |  |
| Birth weight (kg) | 0.353 | 0.246*** | | 0.0637* | 0.0464 | -0.00617 |
|  | (1.21) | (4.54) | | (2.34) | (1.80) | (-0.22) |
| Height (z) t1 |  |  | | 0.777*** | -0.0164 | 0.0175 |
|  |  |  | | (75.69) | (-0.91) | (0.88) |
| Height (z) t2 |  |  | |  | 0.0207 | -0.00234 |
|  |  |  | |  | (1.07) | (-0.11) |
| Cognition (z) t3 |  |  | |  |  | 0.236*** |
|  |  |  | |  |  | (10.31) |
| Prior fetal or child death | -1.231** | 0.108 | | 0.0187 | -0.0493 | -0.0271 |
|  | (-2.64) | (1.29) | | (0.45) | (-1.26) | (-0.62) |
| Breastfeeding |  | -0.0390*** | | 0.00285 | 0.00435* | -0.00442* |
|  |  | (-10.28) | | (1.47) | (2.36) | (-2.16) |
| Constant | 13.72*** | -1.185*** | | -0.735*** | -0.415** | 0.698*** |
|  | (8.72) | (-3.58) | | (-5.18) | (-3.29) | (5.48) |
| Observations | 2410 | 2410 | 2410 | | 2410 | 2410 |

Standard errors in parentheses. ^†^p<0.1 * p<0.05 ** p<0.01 *** p<0.001

¹ Based on whether mother reported knowing about oral contraceptives, injections, IUDs, and Norplant
